# Interactive Virtual Assistant for Health Promotion Among Older Adults With Type 2 Diabetes: The IVAM-ED Randomized Clinical Trial

**DOI:** 10.1001/jamanetworkopen.2025.53508

**Published:** 2026-01-23

**Authors:** Lucas S. Matzenbacher, Frederico Ludwig da Costa, Laura Gomes Boabaid de Barros, Vicenzo Gheno, Isabela Semmelmann Maia, Luiza Machado Blank, Maria Antônia Bertuzzo Brum, Lucas Friedrich Fontoura, Thiago Wendt Viola, Carla Helena Augustin Schwanke, Giovani Gadonski, Janine Alessi, Gabriela Heiden Telo

**Affiliations:** 1 Graduate Program in Medicine and Health Sciences, Pontifical Catholic University of Rio Grande do Sul, Porto Alegre, Rio Grande do Sul, Brazil; 2 Clinical Trials Unit, Moinhos de Vento Hospital, Moinhos de Vento College of Health Sciences, Porto Alegre, Rio Grande do Sul, Brazil; 3 School of Medicine, Pontifical Catholic University of Rio Grande do Sul, Porto Alegre, Rio Grande do Sul, Brazil; 4 Division of Internal Medicine, São Lucas Hospital, Pontifical Catholic University of Rio Grande do Sul, Porto Alegre, Rio Grande do Sul, Brazil; 5 Division of Geriatrics, São Lucas Hospital, Pontifical Catholic University of Rio Grande do Sul, Porto Alegre, Rio Grande do Sul, Brazil; 6 Graduate Program in Biomedical Gerontology, Pontifical Catholic University of Rio Grande do Sul, Porto Alegre, Rio Grande do Sul, Brazil

## Abstract

**Importance:**

Older patients with diabetes often experience high levels of mental distress, which may complicate diabetes management and negatively impact health outcomes.

**Objective:**

To evaluate whether an interactive virtual assistant device built with a behavioral intervention model can improve mental health and diabetes-related outcomes in older adults with diabetes.

**Design, Setting, and Participants:**

This open randomized clinical trial was conducted at an academic medical center in Brazil between June 22, 2023, and February 8, 2024. Participants aged 65 years or older with type 2 diabetes were included.

**Interventions:**

Participants were randomized 1:1 to receive the interactive virtual assistant device (Smart Speaker Echo Dot, 3rd generation [Amazon]) for home use over a 12-week period or to continue with usual care. The device was programmed with a behavioral intervention model aimed at enhancing mental health and diabetes management.

**Main Outcomes and Measures:**

The primary outcome was the between-group mean difference (MD) in mental distress, assessed using the Self-Reporting Questionnaire, in which scores range from 0 to 20, with higher scores indicating greater mental distress. Secondary outcomes included quality of life (assessed using the 36-Item Short Form Health Survey), perceived stress (assessed using the Perceived Stress Scale), adherence to diabetes self-care behaviors (assessed using the Self-Care Inventory Revised questionnaire), and glycemic control (hemoglobin A_1c_). All outcomes were assessed at the 12-week follow-up.

**Results:**

Among 112 participants (mean [SD] age, 72.5 [5.7] years; 71 females [63.4%]), the mean (SD) hemoglobin A_1c_ level was 7.9% (1.5%), and outcome measures were available for 103 participants (52 in the intervention group and 51 in the usual care group). At 12 weeks, the fully adjusted mean (SE) Self-Reporting Questionnaire score was 6.29 (0.44) for participants in the intervention group and 7.75 (0.42) for those in the usual care group, resulting in a fully adjusted MD of −1.46 (95% CI, −2.73 to −0.19; *P* = .02), suggesting a reduction in mental distress among the intervention group. Subgroup analyses showed consistent improvement favoring the intervention across all subgroups. The intervention was also associated with improvements in quality of life (MD, 9.46 [95% CI, 3.65 to 15.26]; *P* = .001), adherence to diabetes self-care behaviors (MD, 3.40 [95% CI, 1.61 to 5.19]; *P* < .001), and glycemic control (MD, −0.48% [95% CI, −0.85 to −0.11]; *P* = .01). No difference was observed regarding perceived stress (MD, −3.00 [95% CI, −6.20 to 0.20]; *P* = .07), and no participants withdrew due to adverse events.

**Conclusions and Relevance:**

In this randomized clinical trial of older adults with diabetes, participants in the smart speaker group showed significant improvements in mental distress, quality of life, diabetes self-care, and glycemic control. These findings suggest that this easily implemented self-management intervention could enhance health outcomes in this population.

**Trial Registration:**

ClinicalTrials.gov Identifier: NCT05329376

## Key Points

**Question:**

Can an interactive virtual assistant device with a behavioral intervention model improve mental health and diabetes management among older individuals with diabetes?

**Findings:**

In this randomized clinical trial involving 112 older adults with type 2 diabetes, participants who used the interactive virtual assistant device experienced significant improvements in mental distress compared with those who received usual care.

**Meaning:**

These findings suggest that an interactive virtual assistant device may enhance mental health outcomes for older individuals with diabetes, serving as a valuable add-on to support and complement existing treatment regimens.

## Teaser

This randomized clinical trial evaluates whether an interactive virtual assistant device with a behavioral intervention model can improve mental health and diabetes management among older adults with diabetes.

## Introduction

One in 5 older individuals lives with type 2 diabetes, representing over 150 million people worldwide.^1,2^ Managing diabetes is complex, especially in at-risk populations such as older adults, which often experience cognitive decline, functional limitations, polypharmacy, and frailty.^3,4^ Additionally, both diabetes and aging increase the risk of mental health disorders, which in turn can further contribute to lower treatment adherence and poorer glycemic control.^5,6,7,8,9^

Developing new strategies to promote mental health and support diabetes management is crucial, and interactive virtual assistants based on artificial intelligence hold promising opportunities to improve lifestyle management and assist with insulin dosing, as demonstrated in previous studies.^10,11^ These devices are more financially accessible than other electronic ones and can provide continuous and personalized support through a user-friendly, voice-activated interface. This is particularly relevant for older adults, as they often face functional limitations and may lack the fine motor skills required to operate more complex devices.

Given the current lack of evidence regarding the use of these devices as a complementary tool to usual care among older adults with type 2 diabetes, we conducted the IVAM-ED (Interactive Virtual Assistant for Mental Health Promotion and Self-Care Management in Elderly with Type 2 Diabetes) randomized clinical trial. The primary objective of this trial was to determine whether an interactive virtual assistant with a voice interface, built with a behavioral intervention model, could improve mental health and reduce psychological distress among older patients with type 2 diabetes. Secondary objectives were to assess its impact on quality of life, perceived stress, adherence to diabetes self-care behaviors, and glycemic control.

## Methods

### Study Design

The IVAM-ED trial was a single-center (São Lucas Hospital, Pontifical Catholic University of Rio Grande do Sul, Porto Alegre, Brazil), open-label, parallel, 2-group randomized clinical trial investigating an interactive virtual assistant device activated by voice for mental health and diabetes-related self-care compared with usual care. The trial protocol and statistical analysis plan were approved by the Research Ethics Committee of Pontifical Catholic University of Rio Grande do Sul and are presented in Supplement 1.^12^ All participants provided written informed consent before enrollment. This study followed the Consolidated Standards of Reporting Trials (CONSORT) reporting guideline.

### Participants

We enrolled older adults (aged ≥65 years) with type 2 diabetes residing in Porto Alegre or the metropolitan region with a Wi-Fi connection at home, who received outpatient care at São Lucas Hospital. Enrollment occurred between June 22, 2023, and February 8, 2024, and the last patients completed the trial in May 2024. A detailed description of the recruitment procedures is provided in the study protocol (Supplement 1).^12^ Key exclusion criteria included having an interactive virtual assistant device and presenting severe hearing or cognitive impairments at the time of enrollment. A full list of the eligibility criteria is provided in eAppendix 1 in Supplement 2.

### Randomization and Blinding

Participants who met the eligibility criteria attended an initial study visit (week −1), in which they signed a consent form and had baseline data collected. At the end of the visit, they were randomly assigned in a 1:1 ratio using a computer-generated simple random sequence without stratification or blocking to receive the interactive virtual assistant device (Smart Speaker Echo Dot, 3rd generation [Amazon]) for home use for 12 weeks or to continue with usual care. All randomization procedures were conducted by an independent researcher (J.A.) and revealed by standard text message at the end of the baseline visit. Participants and investigators were blinded until the end of baseline evaluation. The investigator responsible for supervising statistical analysis remained blinded until all study analyses were completed.

### Interventions

#### Intervention Group (Smart Speaker Group)

Participants assigned to the intervention group received the smart speaker for home use. The device was installed during a home visit by the research team 7 days (±7 days) after the baseline assessment. It was programmed using a standard multicomponent behavioral intervention model, featuring automatic interactions for medication and glucose test reminders, health tips, and educational podcasts to improve diabetes management and mental health. The behavioral intervention was developed by a multidisciplinary diabetes care group specifically for this study and was implemented using the device’s native reminder function for medication and glucose-test alerts, together with a custom Alexa Skill designed to automatically deliver the daily health tips and weekly podcast components of the intervention. A user manual, containing information on the device’s main commands (eg, “Alexa, play music”), was provided to encourage the use of native device functions. Detailed content and frequency of each health tip and podcast episode are available in the study protocol (Supplement 1).^12^

#### Control Group (Usual Care)

Individuals assigned to the usual care group were instructed to maintain their usual care routine. Additionally, they received a booklet with general health information and guidance on accessing a webpage, created by the research team, in which they could listen to the podcast episodes and view the health tips that were automatically provided through the smart speaker to participants in the intervention group. The booklet was provided to standardize the information given to participants in both the intervention and usual care groups.

#### Follow-Up Phone Calls

All participants enrolled in the study received 5 follow-up phone calls, conducted every 2 weeks. The main goal of the calls was to prevent dropouts and monitor adverse events. No motivational interventions were conducted. To ensure equal contact across groups and to minimize potential bias, participants in the usual care group received an additional call at week 0, corresponding to the device installation.

### Study Procedures and Timeline

Participants attended 2 in-person visits at the Clinical Research Center of São Lucas Hospital as part of the study: the baseline evaluation (week −1) and the final evaluation at the end of the intervention period (week 12). Sociodemographic characteristics were assessed at baseline and were self-reported. Outcomes were assessed at both visits by a trained member of the research team (L.S.M., F.L.d.C., L.G.B.d.B., V.G., I.S.M., L.M.B., and M.A.B.B.). The intervention period began at week 0, marked by the device installation (intervention group) or the additional phone call (usual care group). A comprehensive study timeline with specific items evaluated during each visit is provided in the study protocol (Supplement 1).^12^

Diabetes management, including medication adjustments, was provided by participants’ usual health care team and occurred independently of the study procedures. As part of the trial, no additional cointerventions were implemented beyond the predefined study components. Consistent with the pragmatic design of the trial, no motivational strategies or adherence support were delivered to sustain engagement with the smart speaker intervention.

### Outcomes

#### Primary Outcome

The prespecified primary outcome was the between-group mean difference (MD) in mental distress at the 12-week follow-up, accessed using the Brazilian version of the Self-Reporting Questionnaire (SRQ-20).^13^ The SRQ-20 score assesses depression, anxiety, and other common mental health disorder symptoms during the month preceding the evaluation. The total score ranges from 0 to 20, with higher scores indicating greater mental distress.

#### Secondary Outcomes

Prespecified secondary outcomes included the between-group MDs at the 12-week follow-up in quality of life, assessed using the 36-Item Short Form Health Survey, in which scores range from 0 to 100 in each of 8 domains, with higher scores indicating greater quality of life; adherence to diabetes self-care behaviors, assessed using the Self-Care Inventory Revised questionnaire, in which scores range from 11 to 55, with higher scores indicating a higher level of care related to diabetes; perception of stress, assessed using the Perceived Stress Scale, in which scores range from 0 to 56, with lower scores indicating a reduced level of stress; and glycemic control (hemoglobin A_1c_ [HbA_1c_]). Additional secondary outcomes included lipid profile (total cholesterol, high-density lipoprotein cholesterol, low-density lipoprotein cholesterol, and triglyceride levels) and blood pressure (systolic and diastolic). All surveys were previously validated to the study population.^14,15,16^ A detailed description of each outcome is provided in eAppendix 2 in Supplement 2.

### Sample Size

For the primary outcome, the study target sample size was 112 (56 per group). This was calculated using a 2-sample *t* test framework, assuming 90% power, an α level of .05, a 1:1 allocation ratio, a 20% dropout rate, and an anticipated effect size of 0.68 (Cohen *d*). The effect size was based on a similar trial published by Fulmer et al.^17^

### Statistical Analysis

#### Study Outcomes

Primary and secondary end point analyses were conducted including all randomized participants, analyzed as randomized. Missing outcome data at 12 weeks were imputed using multiple imputation by chained equations with 5 imputations, assuming data were missing at random. As all variables were continuous, linear regression models were used for imputation. As the study was pragmatic in nature, adherence to the intervention was not considered for the analyses.

To assess the impact of the intervention, between-groups pairwise comparisons of the final mean of each outcome variable at 12 weeks were conducted using analysis of covariance. Two analysis of covariance models (baseline-adjusted model and fully adjusted model) were performed as prespecified in the statistical analysis plan. Both models included randomization as a fixed factor and the outcome variable as the dependent variable. For the baseline-adjusted model, the baseline value of the corresponding outcome variable was included as a covariate. The fully adjusted model included age, sex, years of education, individual income, and Mini-Mental State Examination score, in which scores range from 0 to 30, with higher scores indicating better cognitive performance, alongside the baseline value of the outcome variable to account for potential imbalances between groups.

Parameter estimates and their corresponding 95% CIs were pooled across the 5 imputed datasets according to Rubin rules and calculated using robust SE estimation. Baseline-adjusted and fully adjusted MDs at 12 weeks, along with their respective 2-sided 95% CIs and *P* values, were reported for each outcome. No adjustments were made for multiple comparisons, and statistical significance was considered at an α level of .05. All statistical analyses were performed using SPSS Statistics, version 27.0.1 (IBM Inc).

The effect size for all end points was reported as standardized MDs. Given the large number of secondary outcomes and the lack of adjustment for multiple comparisons, secondary end points should be considered only for hypothesis generation.

#### Sensitivity Analysis

Seven subgroup analyses were conducted for the primary outcome: sex (male vs female), age (<75 years vs ≥75 years), educational level (primary or less vs secondary or higher), cognitive decline (present vs absent, based on the Mini-Mental State Examination score with education-adjusted cutoffs), insulin use (yes vs no), history of depression (yes vs no), and history of anxiety (yes vs no). Six subgroup analyses were prespecified in the study protocol, and an additional unplanned analysis of history of anxiety was included due to its high prevalence in the sample. The age cutoff was originally set at 80 years, but this was changed to 75 years due to the low number of participants aged 80 years or older. Similarly, the educational-level category was adjusted from graduate to secondary education, given the small number of participants with a graduate degree. Formal comparisons between subgroups were not performed, and *P* values were not reported.

In addition to the subgroup analyses, 2 prespecified sensitivity analyses were performed using different methods for handling missing data: (1) a baseline observation carried forward approach and (2) a complete-case analysis excluding participants with missing outcome data. These analyses aimed to assess the impact of missing data on the study outcomes.

## Results

### Study Participants

**Figure 1.**
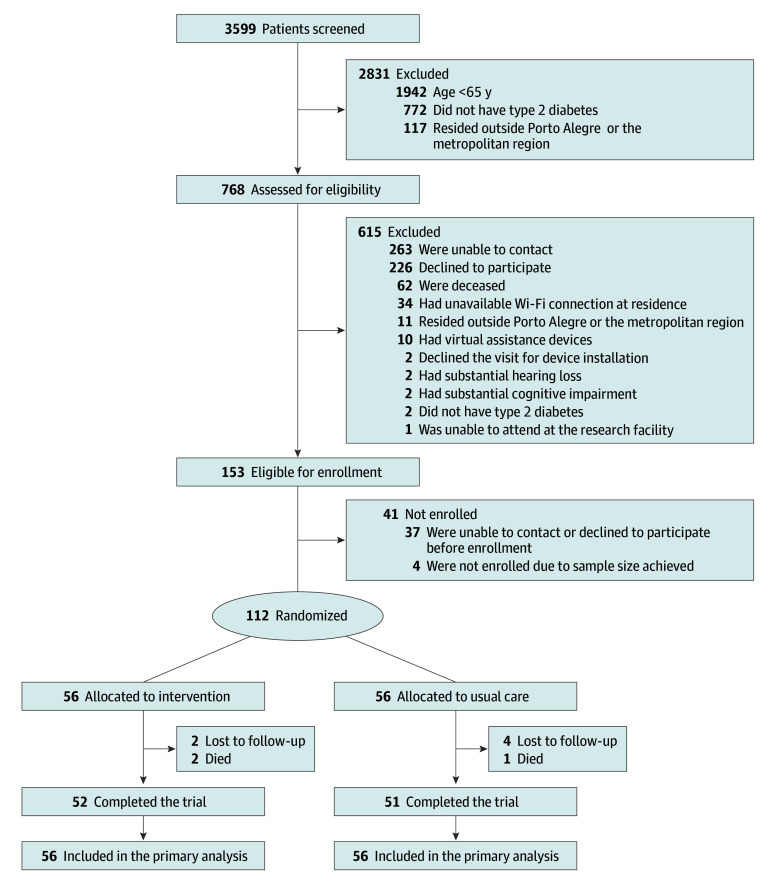
Participant Flow Diagram for the IVAM-ED (Interactive Virtual Assistant for Mental Health Promotion and Self-Care Management in Elderly with Type 2 Diabetes) Trial

Between June 22, 2023, and February 8, 2024, a total of 112 participants (56 per group; mean [SD] age, 72.5 [5.7] years; 71 females [63.4%] and 41 males [36.6%]) were randomized (Figure 1). Overall, 103 participants (92.0%) completed the trial and had the final outcome assessments (52 in the intervention group and 51 in the usual care group). Reasons for nonattendance at the 12-week follow-up included being lost to follow-up (6 participants) and death (3 participants). None of the participants withdrew from the study. Baseline demographic and clinical characteristics were similar across study groups (Table 1 and eTables 1 and 2 in Supplement 2) and between participants with and without missing outcome data (eTable 3 in Supplement 2).

**Table 1.** Demographic and Clinical Characteristics of the Enrolled Participants at Baseline^a^

Characteristic	Usual care group (n = 56)	Smart speaker group (n = 56)
Age, y	72.4 (6.0)	72.6 (5.4)
Weight, kg	76.0 (15.3)	77.1 (15.3)
BMI	30.0 (6.1)	31.2 (6.0)
Sex, No. (%)		
Female	37 (66.1)	34 (60.7)
Male	19 (33.9)	22 (39.3)
Educational level, No. (%)		
No qualification	6 (10.7)	11 (19.6)
Primary education	32 (57.1)	36 (64.3)
Secondary education	16 (28.6)	7 (12.5)
University degree or higher	2 (3.6)	2 (3.6)
Educational level, y^b^	7.5 (3.9)	6.4 (4.2)
Monthly income, No. of Brazilian minimum wages^c^	2.5 (2.7)	2.1 (2.2)
Diabetes duration, y	16.5 (11.1)	17.3 (12.2)
Diabetes complication, No. (%)		
Macrovascular	37 (66.1)	36 (64.3)
Microvascular	28 (50.0)	35 (62.5)
Diabetes medication, No. (%)		
Oral hypoglycemic agents		
Metformin	49 (87.5)	42 (75.0)
Sulfonylureas	17 (30.4)	15 (26.8)
SGLT-2 inhibitors	11 (19.6)	17 (30.4)
DPP-4 inhibitors	5 (8.9)	2 (3.6)
Glitazones	2 (3.6)	0
GLP-1 receptor agonists	1 (1.8)	0
Insulin	28 (50.0)	32 (57.1)
HbA_1c_ level, %	7.8 (1.5)	8.0 (1.6)
HbA_1c_ level, mmol/mol	61.7 (16.0)	63.4 (17.0)
Mental health, No. (%)		
Suicidal thoughts	7 (12.5)	5 (8.9)
Depression	21 (37.5)	19 (33.9)
Anxiety	23 (41.1)	15 (26.8)
Bipolar disorder	2 (3.6)	1 (1.8)
Blood pressure, mm Hg^d^		
Systolic	136.5 (22.0)	135.8 (20.9)
Diastolic	75.4 (10.3)	77.1 (10.6)
Plasma lipid level, mg/dL		
Total cholesterol	177.0 (48.6)	181.1 (48.4)
LDL cholesterol	98.9 (37.9)	101.3 (41.6)
HDL cholesterol	50.7 (13.3)	49.2 (14.7)
Triglycerides	165.0 (90.2)	195.2 (149.2)
MMSE score^e^	24.8 (4.0)	23.5 (4.5)
SRQ-20 score^f^	7.0 (4.7)	7.9 (5.2)
SCI-R score^g^	36.5 (5.9)	35.2 (6.8)
PSS score^h^	23.6 (10.3)	20.6 (10.3)
SF-36 score^i^	50.2 (21.5)	50.9 (22.2)

Abbreviations: BMI, body mass index (calculated as weight in kilograms divided by height in meters squared); DPP-4, dipeptidyl peptidase-4; GLP-1, glucagon-like peptide-1; HbA_1c_, hemoglobin A_1c_; HDL, high-density lipoprotein; LDL, low-density lipoprotein; MMSE, Mini-Mental State Examination; PSS, Perceived Stress Scale; SCI-R, Self-Care Inventory Revised; SF-36, 36-Item Short Form Health Survey; SGLT-2, sodium-glucose cotransporter-2; SRQ-20, Self-Reporting Questionnaire.

SI conversion factors: To convert percentage of total hemoglobin to proportion of total hemoglobin, multiply by 0.01; to convert total, LDL, and HDL levels to millimoles per liter, multiply by 0.0259.

^a^ Data are reported as mean (SD) of participants unless otherwise indicated. All baseline variables were available for all participants.

^b^ Number of complete years of study.

^c^ Each minimum wage corresponds to a monthly income of R$1320.00 (US $257.35 or €237.86, based on the exchange rate at the time of data collection). For reference, a mean income of 2.5 minimum wages corresponds to R$3300.00 (US $643.00 or €595.00) per month, and 2.1 minimum wages corresponds to R$2770.00 (US $540.00 or €500.00) per month.

^d^ Mean of 3 repeated measures of blood pressure with an appropriate cuff considering the arm circumference.

^e^ Scores range from 0 to 30, with higher scores indicating better cognitive performance.

^f^ Scores range from 0 to 20, with higher scores indicating greater mental distress.

^g^ Scores range from 11 to 55, with higher scores indicating a higher level of care related to diabetes.

^h^ Scores range from 0 to 56, with lower scores indicating a reduced level of stress.

^i^ Average of the total score on the 8 domains of the SF-36 questionnaire, ranging from 0 to 100, with higher scores indicating greater quality of life.

The mean (SD) HbA_1c_ level was 7.9% (1.5%) (to convert percentage of total hemoglobin to proportion of total hemoglobin, multiply by 0.01), and the mean (SD) duration of diabetes was 16.9 (11.6) years, with 68.8% of participants living with diabetes for 10 years or more. The socioeconomic level of participants was low, as the mean (SD) individual income was 2.3 (2.4) Brazilian minimum wages per month, and 24.1% of participants had completed secondary education or higher. 

### Mental Distress

The baseline-adjusted mean (SE) SRQ-20 score at the end of the intervention period was 6.39 (0.45) for the smart speaker group and 7.66 (0.43) for the usual care group. Pairwise comparisons between groups at 12 weeks revealed an MD of −1.28 (95% CI, −2.51 to −0.04; *P* = .04) (Table 2). The difference remained significant after adjusting for sociodemographic variables, with a fully adjusted mean (SE) SRQ-20 score of 6.29 (0.44) for participants in the intervention group and 7.75 (0.42) for those in the usual care group (MD, −1.46 [95% CI, −2.73 to −0.19]; *P* = .02), suggesting a reduction in mental distress among participants who used the smart speaker and a positive impact of the intervention on mental health.

**Table 2.** Primary Outcome at 12-Week Follow-Up

SRQ-20 score^a^	Usual care group (n = 56)	Smart speaker group (n = 56)	Mean difference (95% CI)	Effect size^b^	*P* value^c^
At baseline, mean (SD)	7.04 (4.67)	7.89 (5.15)	NA	NA	NA
At 12 w, baseline-adjusted mean (SE)^d^	7.66 (0.43)	6.39 (0.45)	−1.28 (−2.51 to −0.04)	0.41	.04
At 12 w, fully adjusted mean (SE)^e^	7.75 (0.42)	6.29 (0.44)	−1.46 (−2.73 to −0.19)	0.48	.02

Abbreviations: NA, not applicable; SRQ-20, Self-Reporting Questionnaire.

^a^ Total score ranging from 0 to 20 points, with lower scores indicating less mental distress.

^b^ Standardized mean difference.

^c^ Between-group pairwise comparisons using analysis of covariance. The model included all enrolled participants, with missing data replaced using multiple imputation. Statistical significance was considered when *P* < .05.

^d^ Only baseline SRQ-20 scores were included as covariates in the baseline-adjusted model.

^e^ Baseline SRQ-20 score, age, sex, educational level, income, and Mini-Mental State Examination score were included as covariates in the fully adjusted model.

### Quality of Life and Stress

Evaluation of quality of life at 12 weeks showed that participants randomized to the smart speaker group had higher 36-Item Short Form Health Survey scores after the intervention period, with a fully adjusted MD of 9.46 (95% CI, 3.65 to 15.26; *P* = .001), indicating an improvement in quality of life (Table 3). Stress levels, as measured by the Perceived Stress Scale, were not statistically significantly lower in the smart speaker group (fully adjusted MD, −3.00 [95% CI, −6.20 to 0.20]; *P* = .07).

**Table 3.** Secondary Outcomes at 12-Week Follow-Up

Outcome	Usual care group (n = 56)	Smart speaker group (n = 56)	Mean difference (95% CI)	Effect size^a^	*P* value^b^
HbA_1c_					
At baseline, mean (SD), %	7.80 (1.47)	7.95 (1.55)	NA	NA	NA
At 12 w, baseline-adjusted mean (SE), %^c^	8.03 (0.13)	7.56 (0.14)	−0.47 (−0.84 to −0.10)	0.51	.01
At 12 w, fully adjusted mean (SE), %^d^	8.04 (0.13)	7.56 (0.14)	−0.48 (−0.85 to −0.11)	0.52	.01
At baseline, mean (SD), mmol/mol	61.73 (15.91)	63.43 (16.82)	NA	NA	NA
At 12 w, baseline-adjusted mean (SE), mmol/mol^c^	64.31 (1.40)	59.14 (1.50)	−5.17 (−9.21 to −1.12)	0.51	.01
At 12 w, fully adjusted mean (SE), mmol/mol^d^	64.36 (1.45)	59.08 (1.52)	−5.28 (−9.34 to −1.23)	0.52	.01
SCI-R score^e^					
At baseline, mean (SD)	36.54 (5.94)	35.20 (6.83)	NA	NA	NA
At 12 w, baseline-adjusted mean (SE)^c^	36.51 (0.62)	39.68 (0.64)	3.17 (1.39 to 4.96)	0.69	<.001
At 12 w, fully adjusted mean (SE)^d^	36.40 (0.63)	39.80 (0.64)	3.40 (1.61 to 5.19)	0.75	<.001
PSS score^f^					
At baseline, mean (SD)	23.64 (10.27)	20.64 (10.32)	NA	NA	NA
At 12 w, baseline-adjusted mean (SE)^c^	22.03 (1.15)	19.55 (1.20)	−2.47 (−5.63 to 0.68)	0.29	.13
At 12 w, fully adjusted mean (SE)^d^	22.29 (1.15)	19.29 (1.19)	−3.00 (−6.20 to 0.20)	0.36	.07
SF-36 score^g^					
At baseline, mean (SD)	50.16 (21.49)	50.90 (22.15)	NA	NA	NA
At 12 w, baseline-adjusted mean (SE)^c^	49.02 (2.06)	57.21 (2.20)	8.20 (2.47 to 13.93)	0.56	.005
At 12 w, fully adjusted mean (SE)^d^	48.39 (1.97)	57.84 (2.13)	9.46 (3.65 to 15.26)	0.68	.001

Abbreviations: HbA_1c_, hemoglobin A_1c_; NA, not applicable; PSS, Perceived Stress Scale; SCI-R, Self-Care Inventory Revised; SF-36, 36-Item Short Form Health Survey.

SI conversion factor: To convert percentage of total hemoglobin to proportion of total hemoglobin, multiply by 0.01.

^a^ Standardized mean difference.

^b^ Between-group pairwise comparisons using analysis of covariance. The model included all enrolled participants, with missing data replaced using multiple imputation. Statistical significance was considered when *P* < .05.

^c^ Only baseline data for the outcome were included as covariates in the baseline-adjusted model.

^d^ Baseline data for the outcome variable, age, sex, educational level, income, and Mini-Mental State Examination score were included as covariates in the fully adjusted model.

^e^ Total score ranging from 11 to 55, with higher scores indicating a higher level of care related to diabetes.

^f^ Total score ranging from 0 to 56 points, with lower scores indicating a reduced level of stress.

^g^ Average of the total score on the 8 domains of the SF-36 questionnaire, ranging from 0 to 100, with higher scores indicating greater quality of life.

### Diabetes-Related Outcomes, Blood Pressure, and Lipid Profile

At the 12-week follow-up, participants from the smart speaker group presented a higher score on the Self-Care Inventory Revised, with a fully adjusted MD of 3.40 (95% CI, 1.61 to 5.19; *P* < .001), suggesting a greater adherence to diabetes-related self-care behavior (Table 3). They also presented lower HbA_1c_ levels, with a fully adjusted mean (SE) HbA_1c_ level at 12 weeks of 7.56% (0.14%) (59.08 [1.52] mmol/mol) in the intervention group compared with 8.04% (0.13%) (64.36 [1.45] mmol/mol) in the usual care group. The fully adjusted MD was −0.48% (95% CI, −0.85 to −0.11; *P* = .01), indicating a significant reduction in the HbA_1c_ level among participants in the intervention group and better glycemic control (Table 3). No significant differences were observed in blood pressure and levels of total cholesterol, low-density lipoprotein cholesterol, high-density lipoprotein cholesterol, or triglyceride concentrations between the groups (eTable 4 in Supplement 2).

### Sensitivity Analysis

**Figure 2.**
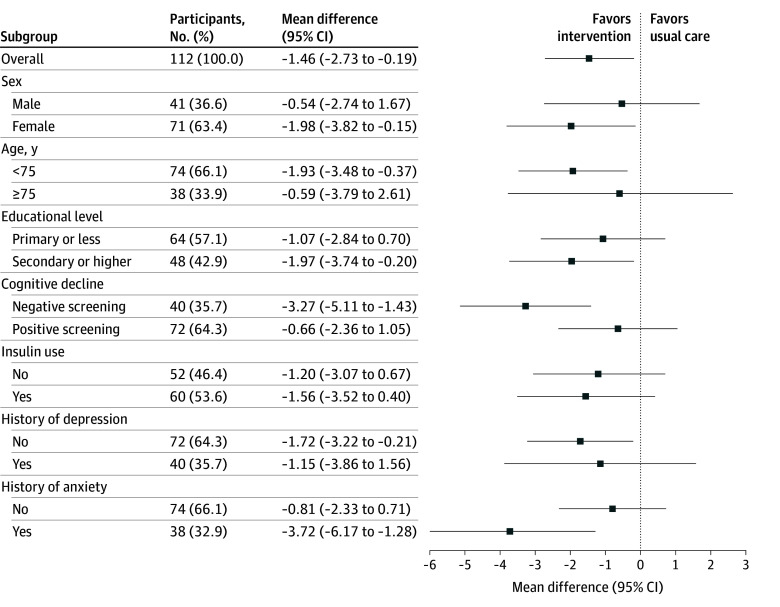
Forest Plot of the Stratified Analysis by Subgroups for the Primary Outcome Mean difference represents between-group pairwise comparisons using an analysis of covariance model with baseline data of the Self-Reporting Questionnaire score (in which scores range from 0 to 20, with higher scores indicating greater mental distress), age, sex, educational level, income, and Mini-Mental State Examination (MMSE) score as covariates. In the stratified analyses by sex, age, educational level, and cognitive decline, the corresponding covariates (age, sex, educational level, and MMSE) were excluded from each model as appropriate. The model included all enrolled participants, with missing data replaced using multiple imputation. No statistical tests were performed to compare specific subgroups, as prespecified in the statistical analysis plan, because no adjustments for multiplicity were applied. Estimates and 95% CIs are reported for hypothesis-generating purposes only.

Stratified fully adjusted analyses for the primary outcomes showed consistent improvements favoring the smart speaker group across all subgroups. Greater MDs were observed among female participants (−1.98 [95% CI, −3.82 to −0.15]), those of younger age (−1.93 [95% CI, −3.48 to −0.37), those without a positive screening for cognitive decline (−3.27 [95% CI, −5.11 to −1.43]), and those with a history of anxiety (−3.72 [95% CI, −6.17 to −1.28]) (Figure 2). However, formal statistical comparisons were not performed between or within subgroups. Sensitivity analyses using different methods for handling missing data yielded similar results to the main analyses carried using multiple imputation (eTables 5 and 6 in Supplement 2).

### Adverse Events

There were no adverse events reported in this study. During follow-up, no participants reported adverse events or severe hypoglycemia episodes in either group. Only 1 participant from the intervention group complained about the frequency of automatic device interactions, but it was not sufficient to cause a substantial disturbance. During the trial, 3 patients died from causes not directly related to mental health or diabetes—1 from stroke, another from urinary sepsis, and a third from heart failure decompensation (eTable 7 in Supplement 2).

## Discussion

In this randomized clinical trial involving 112 older adults with type 2 diabetes, we demonstrated the effectiveness of an interactive virtual assistant, built with a behavioral intervention model, in reducing mental distress compared with usual care. After 12 weeks, participants in the smart speaker group showed significant improvements in mental distress, with a reduction of −1.46 points on the SRQ-20 scale. The intervention was also associated with enhanced quality of life, better adherence to diabetes self-care behaviors, and improved glycemic control, although the impact on secondary outcomes should be interpreted as exploratory given the absence of multiplicity adjustment.

The improvement in mental distress observed in our study supports the hypothesis that smart speakers can provide emotional support for older people with diabetes. In a qualitative study conducted with patients from the intervention group, nearly 30% of participants reported feelings of humanization toward the device.^18^ This perception of the device as quasi-human may have contributed to the observed improvements in mental distress and quality of life in the IVAM-ED trial.

Beyond mental health and quality of life, the intervention was also associated with increased adherence to diabetes self-care behaviors and improved glycemic control, reflected in a 0.48% reduction in the HbA_1c_ level after 12 weeks of intervention. The continuous support and personalized medication reminders may have contributed to better adherence to diabetes management practices, ultimately improving diabetes-related outcomes. Additionally, the improvement in mental health may have positively influenced diabetes self-care behaviors, as reduced mental distress can enhance both motivation and capacity for disease management.^19^

Although consistent improvements were observed across all subgroups, the benefits for the primary outcome appeared to be greater among females, an important finding given that anxiety and mood disorders have consistently been shown to be more prevalent among women.^20,21^ Also, the greater impact observed among those with a previous history of anxiety and the lower impact among those with a history of depression suggest that the improvements may be more closely related to anxiety symptoms, as individuals with depression may be less engaged with the device due to their depressive mood. Additionally, subgroup analyses revealed that participants with higher educational backgrounds, those without cognitive decline, and those under the age of 75 years appeared to benefit more from the intervention. These findings suggest that older adults with lower educational backgrounds, cognitive limitations, or more advanced age may derive fewer benefits from a smart speaker–based intervention, potentially due to greater challenges in adapting to and engaging with the device.

Smart speakers have been proposed to enhance independent living, reduce loneliness, and support patient self-management, with over 50 studies^22^ demonstrating their feasibility and acceptability in health-related settings, although clinical trials evaluating their effectiveness remain scarce. In this context, the IVAM-ED trial provides novel evidence that a smart speaker–based behavioral intervention can improve mental distress and diabetes-related outcomes among older adults with type 2 diabetes. To our knowledge, it is the first randomized study to evaluate the impact of such an intervention on diabetes management in older adults, highlighting its potential clinical relevance while underscoring the need for further research. Future studies with longer follow-up periods, a larger proportion of participants aged 75 years or older, and device modifications to enhance accessibility for those with cognitive limitations or limited educational backgrounds could provide valuable insights into the intervention’s impact across a broader older population.

### Limitations

This study has limitations. First, the nature of the intervention required an open-label design, which could introduce performance, detection, and response bias. Follow-up phone calls were conducted to help mitigate performance bias, and similar dropout rates across study groups support internal validity. Systematic training of the research team was implemented to reduce detection and response bias, and the improvement in HbA_1c_ level, an objective outcome not subject to participant or assessor influence, reinforces the validity of the study’s findings. Second, the intervention period was short, which could have led to an overestimation of the intervention’s effect size, as the impact of behavioral interventions tends to decline with longer follow-up periods. Third, the study’s inclusion criteria requiring a Wi-Fi connection at home may have selected participants more familiar with technology and therefore more likely to interact with the device and benefit from it, although only a small proportion of participants assessed for eligibility lacked Wi-Fi at home. Additionally, our study included participants from a middle-income country, recruited at a single study site, with low income and limited educational background, which may further limit the generalizability of the findings.

## Conclusions

This randomized clinical trial found that an interactive virtual assistant device, built with a behavioral intervention model, improved mental distress among older individuals with type 2 diabetes compared with standard care. Exploratory analyses also suggested improvements in quality of life, adherence to diabetes-related self-care behaviors, and glycemic control. These findings suggest that this easily implemented self-management intervention could significantly enhance health outcomes in this population.

## Notes

Supplement 1.
Study Protocol and Statistical Analysis Plan

Supplement 2. **eAppendix 1.** Eligibility Criteria **eAppendix 2.** Study Outcomes **eTable 1.** Standardized Differences Between Study Groups for Demographic and Clinical Characteristics at Baseline **eTable 2.** Additional Demographic and Clinical Characteristics of the Enrolled Participants **eTable 3.** Baseline Demographic and Clinical Characteristics of Patients With and Without Missing Outcome Data at 12-Week Follow-Up **eTable 4.** Additional Secondary Outcomes at 12-Week Follow-Up **eTable 5.** Sensitivity Analysis of Primary and Secondary Outcomes at 12-Week Follow-Up Excluding Participants With Missing Outcome Data **eTable 6.** Sensitivity Analysis of Primary and Secondary Outcomes at 12-Week Follow-Up Using BOCF for Missing Outcome Data **eTable 7.** Reported Deaths During the Study Period

Supplement 3.
Data Sharing Statement
