# Supplementary material for: Interactive Virtual Assistant for Health Promotion Among Older Adults With Type 2 Diabetes: The IVAM-ED Randomized Clinical Trial
Source: JAMA Netw Open. 2026 Jan 23;9(1):e2553508. doi: 10.1001/jamanetworkopen.2025.53508 (PMC12831153; doi:10.1001/jamanetworkopen.2025.53508)
Supplement: Supplement 1. — Study Protocol and Statistical Analysis Plan [file jamanetwopen-e2553508-s001.pdf]

Study Protocol and Statistical Analysis Plan

Interactive Virtual Assistant for Self-Care Management and Mental Health Promotion in Elderly with Type 2 Diabetes

---

TABLE OF CONTENTS

1. Administrative Information 3
2. Background 4
3. Research question 4
4. Trial Design 4
5. Trial settings and population 4
5.1. Eligibility criteria 5
5.1.1 Inclusion criteria 5
5.1.2 Exclusion criteria 5
5.2. Baseline evaluation and informed consent 5
5.3. Randomization and blinding 6
6. Trial interventions 6
6.1. Intervention group (Alexa's group) 6
6.2. Control group (usual care) 11
6.3. Follow up phone calls 12
7. Outcome definition 12
7.1. Primary outcome 12
7.2 Secondary outcomes 12
8. Outcome assessors 13
9. Study assessments and timeline 13
9.1. Data collected 15
10. Missing appointments, early withdrawal, and loss of follow-up of participants 16
11. Sample size 17
12. Statistical analysis plan 17
12.1 Descriptive statistics and baseline data 17
12.2. Primary and secondary outcome analysis 17
12.3. Missing data 18

12.4. Adjustment for covariates 19
12.5. Sensitivity and subgroup analysis 19
12.6. Adjustment for multiple testing 19
12.7. Confidence intervals, p-value and reporting conventions 19
12.8. Adherence to the intervention 20
12.9. Statistical software 20
13. Adverse event monitoring 20
14. Data management and confidentiality 20
15. Funding 21
16. Institutional review board approval 21
17. Monitoring and additional information 21
17.1. Composition of the coordinating center and trial steering committee 21
17.2. Trial Sponsor 21
17.3. Frequency and plans for auditing trial conduct 21
17.4. Communication of important protocol modifications 22
17.5. Declaration of interests 22
18. Trial status 22
19. List of abbreviations 22
20. References 23
Appendix A: SPIRIT Checklist 25
Appendix B: Description of the 28 daily messages repeated every 4 weeks in Brazilian Portuguese 30
Appendix C: Description of the 7 sets of 14 messages repeated every week translated in Brazilian Portuguese 33

1. Administrative Information

TRIAL FULL TITLE	Interactive Virtual Assistant for Self-Care Management and Mental Health Promotion in Elderly with Type 2 Diabetes
TRIAL REGISTRATION	NCT05329376
COORDINATOR AND SENIOR INVESTIGATOR	Gabriela Heiden Telo
LOCATION	Porto Alegre, Rio Grande do Sul, Brazil
MAIN CENTER	São Lucas Hospital, Pontifícia Universidade Católica do Rio Grande do Sul, Porto Alegre - RS, Brazil
SPONSOR	Gabriela Heiden Telo (gabriela.telo@pucrs.br) as part of the Diabetes and Endocrinology Research Group at the Pontifícia Universidade Católica do Rio Grande do Sul
PROTOCOL AUTHOR(s)	Lucas Strassburger Matzenbacher and Frederico Ludwig da Costa
PROTOCOL DATE	April, 2023
PROTOCOL VERSION	3.0
SAP AUTHOR(s)	Lucas Strassburger Matzenbacher
SAP DATE	June, 2023
SAP VERSION	1.0
IRB APPROVAL NUMBER	5274307

2. Background

The use of voice-activated technology and similar devices for assisting elderly individuals and those with type 2 diabetes has garnered widespread international interest. While substantial efforts have been made to develop these technologies for healthcare enhancement, their adoption remains limited, highlighting the need for more comprehensive initiatives to fully utilize their potential.

Despite many possible applications, evidence on the efficacy of these technologies in the elderly population is limited. Therefore, we developed a model of behavioral intervention delivered through the Smart Speaker Echo Dot 3rd Gen (Amazon Alexa) to promote knowledge and skills necessary for mental health promotion and diabetes self-management in this population.

3. Research question

For elderly individuals with type 2 diabetes, what is the effect of an interactive virtual assistance system activated by voice delivered through Smart Speaker EchoDot 3rd Gen (Amazon Echo®) in:

1. Mental health;
2. Quality of life;
3. Diabetes self-care behavior;
4. Glycemic profile;
5. Blood pressure; and
6. Lipid profile.

4. Trial Design

This is a single-center, pragmatic, volunteer-driver, registry-based, parallel two-arm (intervention-to-control group ratio=1:1) open superiority randomized clinical trial. We followed the Standard Protocol Items: Recommendations for Interventional Trial (SPIRIT) 2013 guideline (1) when writing this protocol and the detailed SPIRIT checklist is available in Appendix A.

5. Trial settings and population

This study is a single-center trial, and all procedures will be conducted at the Center for Clinical Research of São Lucas Hospital (HSL), in Southern Brazil, under the supervision

of the Pontifícia Universidade Católica do Rio Grande do Sul (PUCRS). Participants will be recruited through two different strategies:

1. Screening of records from the outpatient clinics of HSL: Records of the outpatient clinics of HSL will be screened by a trained recruiter who will collect basic information about the inclusion criteria (date of birth, type 2 diabetes diagnosis and city of residence). Individuals who meet the basic inclusion criteria will receive a phone call for explanation about the study. Those who express interest in participating will undergo an assessment to determine eligibility.

2. Social media advertising: An electronic form via Google Forms® will be advertised via Instagram®. Interested volunteers who meet the basic inclusion criteria will receive a phone call to provide additional information about the study and to assess eligibility.

Subjects who do not respond to the initial phone call will be contacted through WhatsApp® message and will receive three phone call attempts at three different times, for a total of nine attempts. If there is no response after a total of nine unsuccessful contact attempts, the subject will be considered unreachable and ineligible for the study.

5.1. Eligibility criteria

5.1.1 Inclusion criteria

- Being 65 years of age or older;
- Having a diagnosis of type 2 diabetes;
- Residing in Porto Alegre or metropolitan region;
- Presenting availability of Wi-Fi connection at home; and
- Presenting availability to participate in the proposed face-to-face evaluation and accept receiving one visit for installation of the device.

5.1.2 Exclusion criteria

- Having an interactive virtual home assistance device at the time of enrollment;
- Having cognitive impairments or severe hearing impairments that prevent adequate interaction with outcome assessors and the application of follow-up questionnaires; or
- Residing in regions of difficult access.

5.2. Baseline evaluation and informed consent

Subjects who meet the eligibility criteria will be considered eligible for randomization and will have a baseline evaluation scheduled. Prior to collecting any data, the outcome

assessor will obtain informed consent from participants and only those who provide it will proceed with the baseline evaluation and randomization.

5.3. Randomization and blinding

Individuals who provide informed consent will undergo randomization through a 1:1 randomization sequence generated using Research Randomizer software (https://www.randomizer.org/). To ensure proper implementation, all aspects of the randomization process, including sequence generation and result disclosure, will be carried out by an independent researcher who will not be related to the study recruitment and outcome assessments. The randomization result will be disclosed only after the end of the baseline evaluation. Therefore, participants and outcome assessors will be blinded only in the baseline evaluation. Additionally, researchers responsible for performing statistical analysis will also be blinded. To ensure this, before extracting the data from the database, random codes for encoding the study groups will be generated by the same independent researcher responsible for allocation. The disclosure of group assignments will only occur after all analyses have been completed.

6. Trial interventions

6.1. Intervention group (Alexa's group)

Individuals assigned to the intervention group will receive the Smart Speaker EchoDot 3rd Gen (Amazon Echo®) device (i.e., Alexa) for home use, installed during a home visit following the baseline evaluation. The device will be programmed using a standard model developed by the research team, applied to each patient. The proposed model involves automatic interactions between the device and the patient, comprising:

1. Medication reminders: The device will be programmed to automatically issue reminders at specified times for patients to take their medications and insulin, following the medical prescription provided by the attending physician.
2. Glucose test reminders: Automatic reminders for glucose testing will also be programmed, aligning with each patient's individual testing frequency and routine.
3. Educational health tips: At two pre-defined times during the day, set in consultation with the patient during installation, the device will emit a sound alert and deliver daily flash briefings centered on health education messages. These briefings will include a greeting message, three messages related to health education, and a farewell message. The health education-related messages

comprise a total of 42 unique messages developed by the research team, divided into two groups:

a. 28 messages that will be repeated every 4 weeks, one message each day, so that each message will be played three times over the 12-week study period. A detailed version of the phrases is available in Table 1 (English, translated) and Appendix B (original version in Brazilian Portuguese).
b. 14 messages divided into 7 sets of 2 messages that will be repeated every week, two messages each day, so that each message will be played 12 times throughout the study. A detailed version of the phrases is available in Table 2 (English, translated) and Appendix C (original version in Brazilian Portuguese).

4. Weekly educational podcasts: The device will be programmed to play 12 weekly educational podcast episodes, each lasting 5 minutes, recorded by the research team. These episodes cover four key themes: physical exercise, diabetes self-care, mental health, and healthy eating habits, with three episodes dedicated to each theme. A detailed description of each episode is provided in Table 3. Each weekly episode will be automatically played twice during the week, at times pre-defined by the patient during the device installation.
5. Good morning and good night routine: The good morning routine activates when the patient says "Alexa, good morning," delivering the weather forecast for the patient's region. Similarly, the good night routine is initiated by the patient saying "Alexa, good night," playing a playlist of low-volume music.

In addition to the pre-determined programmed model, the individual will also receive a user manual for the device containing information about the main device commands (for example, “Alexa, play music”), common possible errors and their solutions, and a list of skills (for example, religious, games, culture, stories, and trivia) available for use on the device to encourage the use of additional functions based on the patient's. If there is any issue with the device, an internet error occurs, or the device remains offline, a new home visit will be scheduled to correct the problem, and the need for a new contact will be recorded.

Table 1. Description of the 28 daily messages repeated every 4 weeks translated to English

No	Content
1	Take some time for yourself today. Perhaps go read a book, magazine, or newspaper.
2	Do an act of kindness for yourself today; go and take a relaxing bath or have a hot cup of tea now.
3	It's important to have moments of relaxation and leisure activities in your daily routine. Try to find some time in your day to do something you enjoy.
4	Maintaining contact with friends and family, including through regular phone calls, is important. How about calling a loved one now?
5	How about challenging your mind by solving memory problems? I can help you with that through a quiz. If you're interested, just say, "Alexa, open My Quiz."
6	Enjoy the early morning or late afternoon sun to get some vitamin D.
7	How about taking a break now and revisiting old photos of special moments? Reliving positive memories is important and can be uplifting.
8	Consider watching a movie or TV show that brings you joy or that you like.
9	Why not try a healthy cooking recipe today?
10	When eating, opt for whole foods such as whole grain bread and cereals instead of refined carbohydrates.
11	How about challenging yourself to increase your consumption of vegetables like lettuce, kale, and spinach this week? These are fiber-rich and nutrient-rich foods that can help with diabetes management.
12	It's important to limit the consumption of processed foods like snacks and frozen meals.
13	You can reduce the salt in your food by using natural seasonings like herbs, spices, and black pepper. How about trying to make a dish with less salt today?

14	You can replace sugary desserts with healthier options like fresh fruits or plain yogurt.
15	Eat small portions of food at each meal and avoid eating large quantities at once.
16	Have balanced meals, including a source of protein or meat, vegetables, few carbohydrates, and low fats.
17	Read food labels and avoid those that contain a lot of sugars and unhealthy ingredients.
18	Whenever possible, cook your meals at home to have control over the ingredients and preparation.
19	Whenever you check your blood glucose level or have a hypoglycemia episode, write it down in a notebook with the date and value.
20	If you have a hypoglycemia episode, remember that just a tablespoon of sugar is enough to reverse it. Be careful, as excess sugar can also be a problem.
21	Have an emergency plan for hypoglycemia, and remember to take something to eat when you leave home.
22	Maintain an adequate stock of diabetes-related medications and supplies, such as glucose test strips, medications, and insulin needles, to avoid interruptions in your treatment.
23	How about learning relaxation techniques like deep breathing and meditation to cope with stress? I can help you with that; just ask, "Alexa, open the meditation skill."
24	Sometimes, it's normal to feel that having diabetes is a burden, and these emotions are part of the process of learning to cope with the disease. Pay attention to your mental and emotional health.
25	Diabetes is a manageable condition; with proper treatment, you can keep your blood glucose levels in check and lead a good and normal life.
26	Seek support from family, friends, and support groups to share experiences, tips, and challenges related to diabetes treatment. Having people who understand and support you can make a big difference.
27	The goal of diabetes treatment is to improve your quality of life and overall well-being. Focus on taking care of yourself and making healthy choices that bring long-term benefits to your life.

28	Even with a diabetes diagnosis, you don't have to give up all the things you love or the foods you enjoy.

Table 2. Description of the 7 sets of 14 messages repeated every week translated to English

No	Content
1	If possible, engage in a stretching session.
	Engaging in moderate-intensity physical activities for 30 minutes a day is enough to stop being sedentary. These 30 minutes can be divided into 10-minute periods. Always strive to stay active.
2	The most common symptoms of hypoglycemia are: tremors, dizziness, excessive sweating, fainting. Always remember to pay attention to the presence of these symptoms.
	Make sure to use the correct insulin doses and always administer them at the time recommended by your medical team.
3	Remember to take care of your feet, be careful not to injure them, and always check for the presence of lesions or wounds. Take care of your footwear and always make sure they are suitable for use.
	Maintain regular medical check-ups, perform your blood glucose tests as directed by your medical team, and make sure to use prescribed medications correctly.
4	In addition, reducing the use of electronic devices with screens a few hours before bedtime is important. All of these actions will help you sleep better.
	Avoid drinking coffee and mate (chimarrão) after 5 PM; these beverages contain caffeine and can disrupt your sleep.
5	Remember to drink water regularly.
	Avoid naps after 5 PM, as it may make it easier to sleep at night.
6	Feeling lonely? How about sending a message or calling a friend or family member now?
	Remember to set achievable daily goals and celebrate small achievements.
7	Shall we meditate now? If you're interested, just ask for the meditation skill.
	Listening to music you enjoy can boost your spirits and well-being. I can play any music for you, just ask: "Alexa, play music."

Table 3. Description of the content featured in the 12 podcast episodes played weekly by the device

Subject	Episode	Description
Physical Exercise	1	Benefits of physical exercise: glycemic control, weight loss, pain reduction, improved mental health and overall quality of life improvement
	2	Weekly goals for physical exercise
	3	Appropriate exercise modalities for seniors and strategies to achieve physical exercise goals
Diabetes Selfcare	1	Foot care and fall prevention.
	2	Glycemic control and hypoglycemia management
	3	Strategies to improve diabetes-related self-care
Mental Health	1	How sleeping impacts on mental health, quality of life, sleep hygiene and habits that improve its quality
	2	Anxiety and depression management and importance of maintaining a social circle
	3	Reflections on aging and the process of the finiteness of life
Healthy Eating Habits	1	Glycemic index and benefits of including fiber and protein in the diet
	2	Strategies to achieve a balanced diet and make better food choices
	3	How to organize a shopping list and identify processed foods to be avoided

6.2. Control group (usual care)

Patients assigned to the control group will be instructed to maintain their usual healthcare routine. Additionally, they will receive a booklet with general information and a QR code linking to a website developed by the research team. This website will provide access to podcast episodes and the phrases automatically delivered via the Alexa device to participants in the intervention group.

6.3. Follow up phone calls

Follow-up of participants will be maintained through five follow-up phone calls, conducted every two weeks. Additionally, participants from the control group will receive an extra phone call, equivalent to the device installation, to ensure equivalent contact in both groups. A detailed timeline for these calls is provided in the following sections. The purpose of the follow-up phone calls is to ensure ongoing monitoring, and no behavioral or motivational interventions will be conducted during them.

7. Outcome definition

This section will present the outcomes investigated to answer the study research question described on section 3. All questionnaires and scores used were previously validated to the population and language of our study (2-5).

7.1. Primary outcome

- **Mental distress:** Depression, anxiety, and other common mental health disorder symptoms will be assessed at baseline and after 12 weeks using the Brazilian Portuguese version of the Self Reporting Questionnaire 20 (SRQ-20). The outcome will be presented as a total score ranging from 0 to 20 points, with higher scores indicating greater mental distress.

7.2 Secondary outcomes

- **Glycemic control:** Glycated hemoglobin (HbA1c) dosage performed using high-performance liquid chromatography at baseline and 12 weeks will be considered for glycemic control evaluation. HbA1c levels will be presented as a continuous variable in percentage and mmol/mol.
- **Adherence to diabetes self-care behaviors:** Adherence will be assessed at baseline and after 12 weeks using the Brazilian Portuguese version of the Self-Care Inventory Revised (SCI-R) questionnaire, which reflects the follow-up of treatment recommendations and lifestyle habits related to diabetes care in the 2 months prior to the application. The outcome will be presented as a total score ranging from 11 to 55 points, with higher scores indicating a higher level of care.
- **Quality of Life:** Quality of Life will be assessed at baseline and after 12 weeks using the Brazilian Portuguese version of 36-Item Short Form Health Survey (SF-

36) questionnaire. It evaluates the quality of life across eight domains: functional capacity, limitation due to physical aspects, pain, general health status, vitality, social aspects, emotional aspects, and mental health. The outcome will be presented as an overall score (mean of the score in the eight domains) ranging from 0 to 100, with higher scores indicating lower quality of life.

- **Perception of stress:** Stress will be assessed at baseline and after 12 weeks using the Brazilian version of the Perceived Stress Scale (PSS) questionnaire. The outcome will be presented as a total score ranging from 0 to 56 points, with higher scores indicating a greater level of stress
- **Blood pressure:** Systolic and diastolic blood pressure will be assessed at baseline and at week 12 using appropriate sphygmomanometer according to the circumference of the arm. Three measures will be made for each subject and the mean of the three repeated measures will be considered as the outcome.
- **Lipid profile:** Total cholesterol, HDL-c, LDL-c and triglycerides dosage in mg/dL will be considered for the lipid profile outcome

8. Outcome assessors

Specific members of the research team will be responsible for conducting all study visits and follow-up phone calls, as well as screening and recruiting participants. To minimize bias, all team members will receive formal training and will be instructed to adhere to the standard operational protocols before the trial's initiation.

9. Study assessments and timeline

All study participants will be expected to attend two in-person follow-up visits: one at baseline (week -1) and another at the final of the intervention period (week 12). Additionally, participants in the intervention group will receive a home visit (week 0) for the installation of the device, while participants in the control group will receive an extra follow-up phone call during the same timeframe to ensure equivalent contact. A detailed timeline is available in Figure 01. Specific items to be evaluated during each appointment, including duration of visits and assessment time windows, as well as the logistics of the follow-up phone calls are available at Table 04 and 05, respectively.

Figure 1. SPIRIT Figure - Schedule of enrolment, interventions, and assessments

	STUDY PERIOD							
	Enrolment	Allocation	Post-allocation					Close-out
TIMEPOINT**	*-1 w*	0 w	*2 w*	*4 w*	*6 w*	*8 w*	*10 w*	*12 w*
**ENROLMENT:**								
Eligibility screen	X							
Informed consent	X							
Randomized by independent person	X							
Allocation		X						
**INTERVENTIONS:**								
Intervention group: Smart Speaker EchoDot 3rd Gen		—	—	—	—	—	—	—
Control group: usual care		—	—	—	—	—	—	—
Follow up phone calls			X	X	X	X	X	
**ASSESSMENTS:**								
Demographic information and baseline data	X							
SRQ-20 (primary outcome)	X							X
SF-36	X							X
SCI-R	X							X
PSS	X							X
Blood pressure	X							X
Glycated hemoglobin (HbA1c)	X							X
Lipid profile	X							X

Table 4: Logistic and assessments of each visit during the study period

Visit	Baseline Evaluation (week -1)	Installation of the device* (week 0)	Final Evaluation (week 12)
Predetermined duration of visit (minutes)	120	15	60
Protocol assessment time windows (days)	NA	±7	±7
Identification data	X		
Clinical interview and baseline evaluation	X		
Clinical evaluation	X		X
Laboratory evaluation	X		X
Medication use	X		X
Installation of device		X	

*This applies only to participants from the intervention group. Participants in the control group will receive an additional phone call in week 0 to ensure equivalency. NA refers to not applied.

Table 5. Logistic of phone calls during the study period

Phone call	Week of study
Phone call 0*	Week 0
Phone call 1	Week 2
Phone call 2	Week 4
Phone call 3	Week 6
Phone call 4	Week 8
Phone call 5	Week 10

*Only for participants from the control group. Equivalent to installation of the device.

9.1. Data collected

Identification data: Full name, medical record number, phone number, address, and email address.

Clinical interview and baseline evaluation: Sociodemographic information, comorbidities and personal medical history, daily schedule, mini-mental state examination (MMSE) score,

up and go test, sit-to-stand test, usual gait speed test, height, hip and calf circumference, Katz activities of daily living index score, and Lawton instrumental activities of daily living scale

Clinical evaluation: Blood pressure (three repeated measures), handgrip strength (three repeated measures in the non-dominant arm), body weight, and score on the Brazilian version of the following:

- SRQ-20
- SF-36
- SCI-R
- PSS

Laboratory evaluation: Glycated hemoglobin, fasting plasma glucose, total cholesterol, HDL-c, LDL-c, triglycerides, serum creatinine, and albumin and creatinine (urine sample)

Medication use: Name and daily dosage of medications, vitamins and nutritional supplements, if in use

Installation of the device: During installation of the device no data is collected.

Follow-up phone calls: General information about well-being and adverse events

10. Missing appointments, early withdrawal, and loss of follow-up of participants

In order to reduce the number of missed appointments, a standardized message will be sent 1 day prior to the scheduled appointment to remember and confirm attendance. If a participant misses an appointment, they will be contacted by phone call to reschedule. To minimize dropouts and missing data, all eligible participants will be informed of the importance of attending follow-up visits during the baseline visit and in every follow-up phone call. If a participant requests early withdrawal from the study, they will be asked to attend to final evaluation to collect endpoint measurements.

Before considering a participant as unresponsive and proceeding to exclude them from the study due to a lack of contact we will conduct 9 phone call attempts (three phone calls at three different moments), in addition to attempts to contact them through WhatsApp messages. Also, three Smart Speaker EchoDot 3rd Gen (Amazon Echo®) devices will be randomly awarded as incentives among participants who successfully complete the study. This strategy aims to encourage participants to provide complete data, thus improving the overall data quality of the study.

11. Sample size

Sample size calculations were performed using the Statistics and Sample Size Pro software (6). With an alpha level of 0.05, a power of 90%, and an effect size of 0.68 (Cohen's d), it was estimated that 94 subjects (47 per group) would be required to assess mean differences in the primary outcome. Assuming a 20% dropout rate, we plan to include 112 subjects (56 per group) in the study. The effect size of 0.68 was based on the randomized controlled trial published by Fulmer R et al. (7), which evaluated the efficacy of using psychological artificial intelligence software to reduce self-identified symptoms of depression and anxiety.

12. Statistical analysis plan

This document serves as the formal statistical analysis plan (SAP) (version 1.0) for the trial. The detailed SAP was elaborated following the guidance published by Gamble et al. (2017)(8).

12.1 Descriptive statistics and baseline data

A consolidated standards of reporting trials (CONSORT) style flow diagram will present the number of patients screened and all reasons for exclusions prior to randomization (9). Demographic and socioeconomic information (including age, sex, race, socioeconomic status, and educational level), baseline clinical characteristics (such as body mass index [BMI], glycated hemoglobin, systolic and diastolic blood pressure, insulin usage, smoking status, alcohol consumption, comorbidities, performance on up and go, gait speed, and sit and stand tests, and others), as well as other variables, will be described by arm and overall. Categorical variables will be described as frequency (percentage). Continuous variables will be described as mean ± standard deviation (SD) or median and interquartile range. Hypothesis testing for differences in baseline characteristics between the intervention and control arms will not be performed, in accordance with the CONSORT guideline.

12.2. Primary and secondary outcome analysis

All analysis will be performed after the end of the trial and no interim analysis will be performed. The analysis of all study outcomes will be carried out using analysis of covariance (ANCOVA), as all the outcomes are quantitative. This approach will allow a comparison of means between groups, while also considering potential variations in covariates. As we will compare the means at the end of the study, rather than deltas

(differences from baseline), the model will include baseline data of the outcome variable as a covariate. This adjustment allows us to account for the initial variation in scores among the groups before assessing any potential intervention effects.

All analysis will be performed using the intention-to-treat approach, including all subjects who were randomized. Per protocol analysis will only be performed as a sensitivity analysis, as described in the section 12.3. Normality tests for outcome data will not be performed. Instead, we will conduct visual histogram and QQ plot analyses. If distributional assumptions are in doubt, we will perform a logarithmic transformation of the data before analysis.

12.3. Missing data

The imputation of missing data will be handled following the European Medicines Agency Guideline on Missing Data in Confirmatory Clinical Trials (10). The overall rate of missing data at week 12 is expected to be no more than 20% and the frequency and reasons for missing data are expected to be missing at random (MAR). However, the possibility of missing not at random (MNAR) cannot be excluded, as we believe that three factors may influence the missing data:

1. Participants less familiar with technology may have a higher tendency to dropout and might experience reduced benefits due to a higher likelihood of non-adherence to the intervention.

2. Participants from the usual care group may have a higher tendency to discontinue participation due to not receiving the device as they expected.

3. Older participants, who are less likely to experience intervention-related benefits, may be more likely to miss the final evaluation due to potential mobility challenges and a greater reliance on caregiver assistance to attend the consultation.

Therefore, to explore the potential impact of missing data in different scenarios, promoting a better understanding of how different methods of handling missing data impact our results considering the challenge of handling missing data within the possibility of MNAR assumption, three distinct imputation strategies will be employed to assess the impact of missing data on our results:

1. *Multiple imputation with baseline data correction:* we will perform multiple imputation of missing data correcting for the baseline data. This correction aims to mitigate potential bias that could be introduced by missing data imputation using multiple imputation under a possible MNAR assumption.

2. Baseline Observation Carried Forward (BOCF): given our anticipation and belief that both control and intervention subjects are likely to exhibit improvement regarding the baseline parameters, we will adopt the BOCF technique as a sensitivity analysis. By carrying forward the baseline observation, our analysis will be conducted within a more conservative scenario that tends toward the null hypothesis, reducing the probability of type 1 error related to missing data imputation.

3. Exclusion of participants with missing data: furthermore, we will conduct a sensitivity analysis considering only the trial completers. As this analysis has the potential to introduce selection bias, the results will be treated as hypothesis generation as subjects with missing data may differ in certain aspects from those without missing data.

12.4. Adjustment for covariates

For adjustment, age, sex, mini-mental state examination score, education, income, and baseline data of the outcome variable will be included in the ANCOVA model as covariates. Additionally, other variables that were found to be imbalanced between groups at baseline will also be included into the analysis.

12.5. Sensitivity and subgroup analysis

Sensitivity analysis will be conducted to assess the impact of missing data as described above. Also, pre-specified subgroup analysis will be performed: age (<80 vs. ≥ 80 years old), education level (graduate vs. undergraduate), sex (male vs. female), insulin usage (yes vs. no), history of depression (yes vs. no) and cognitive impairment (yes vs. no, accordingly to MMSE score adjusted for education level). Due to the increased risk of type I and type II error and reduced power, only subgroup analysis for primary outcome will be performed.

12.6. Adjustment for multiple testing

No adjustment for multiple testing will be performed. Therefore, all outcomes rather than primary outcome will be considered hypothesis generation and p-value will not be reported.

12.7. Confidence intervals, p-value and reporting conventions

Statistical significance level for primary outcome will be at the 0.05 level ($\alpha = 0.05$). 95% confidence intervals for all study outcomes, as well as for all subgroup and sensitivity

analysis will be reported. P-value values ≥0.001 will be reported to 3 decimal places; values <0.001 will be reported as “<0.001”. Mean and SD values will be reported using one decimal place greater than the original data. Median and quantiles will use the same number of decimal places as the original data. Subjects who attend to baseline evaluation and final evaluation visits will be considered trial completers.

12.8. Adherence to the intervention

As a pragmatic trial aimed to evaluate the intervention effectiveness in a setting closer to a real-world scenario, the evaluation of outcomes will not consider patients adherence to the proposed intervention, maintaining the integrity of the intention-to-treat analysis.

12.9. Statistical software

The statistical software RStudio (version 4.3.1 or above) and IBM® SPSS Statistics (version 27.0.1 or above) will be used for all the analyses

13. Adverse event monitoring

No adverse events are expected to occur as a result of the intervention. However, there is potential for participants to experience stress related to the intervention, which will be recorded as part of the primary outcome of the study. Furthermore, given that the study involves blood collection, there is a possibility of harm not directly linked to the intervention. Anticipated adverse effects such as hematoma and local pain will be monitored. Any serious adverse effects beyond these will be recorded and reported, although such occurrences are considered highly unlikely. Additionally, during the follow-up phone calls, participants will be asked about their well-being and if they have experienced any changes or symptoms since the start of the study. This information will be documented to ensure the ongoing safety and monitoring of the participants, even though specific adverse event classification will not be applicable in this study.

14. Data management and confidentiality

Data will be collected in physical records by the study outcome assessors and stored in a two-level locker room in the research facility. All the data will be entered into a database in the REDCap platform. Outcome variables will be entered in duplicate to ensure data quality. To maintain confidentiality, study participants will be identified using an

identification number (ID), and only the main investigators of the study will have access to identification information (i.e., names) and complete data sets.

15. Funding

This study received funding from the Brazilian National Council for Scientific and Technological Development (CNPq) through the CNPq/MCTI/FNDCT 18/2021 grant. Additionally, the study receives financial support from the Coordination for the Improvement of Higher Education Personnel - Braizil (CAPES), finance code 001. The funders are not involved in the conceptualization, implementation, or analysis of the study. Our research has no affiliation with Amazon, and we do not receive any funding from the company. The Smart Speaker EchoDot 3rd Gen (Amazon Echo®) devices will be acquired with CNPq funding.

16. Institutional review board approval

This study protocol was submitted for approval by the Institutional Review Board (IRB) of Pontifícia Universidade Católica do Rio Grande do Sul. No study procedures were conducted prior to IRB approval.

17. Monitoring and additional information

17.1. Composition of the coordinating center and trial steering committee

The Diabetes and Endocrinology Research Group at the Pontifical Catholic University of Rio Grande do Sul will serve as the coordinating center and trial steering committee for this study.

17.2. Trial Sponsor

The senior investigator of the trial serves as the Sponsor-investigator for this study. They are responsible for ensuring that research ethics principles are followed, supervision, study design, arranging the financing, data collection, statistical analysis, report writing, and the decision to submit for publication.

17.3. Frequency and plans for auditing trial conduct

No formal audits are planned for this study. Instead, the Sponsor-investigator will ensure that the study is implemented in accordance with the protocol and will promptly inform the Research Ethics Committee of Pontifícia Universidade Católica do Rio Grande do Sul of any potential risks that arise during the study. The trial steering committee will

convene at least once a month to assess the progress of the study. Due to the short duration of the trial, the small sample size, and the minimal risk to participants, a separate data monitoring committee was not proposed.

17.4. Communication of important protocol modifications

If necessary, additional protocol modifications will be reported to the Research Ethics Committee of Pontifícia Universidade Católica do Rio Grande do Sul and made publicly available by updating the ClinicalTrials.gov registry by the Sponsor-Investigator. Additionally, any deviations from this study protocol will be reported and justified in all publications related to the study.

17.5. Declaration of interests

The authors declare that they have no competing interests.

18. Trial status

This is a protocol (version 3.0, June 2023) for an ongoing trial that started recruitment on June, 2023.

19. List of abbreviations

SPIRIT	Standard Protocol Items: Recommendations for Interventional Trials
HSL	Hospital São Lucas
PUCRS	Pontifícia Universidade Católica do Rio Grande do Sul
SRQ-20	Self Reporting Questionnaire 20
SCI-R	Self-Care Inventory Revised
SF-36	36-Item Short Form Health Survey
PSS	Perceived Stress Scale
HDL-c	high-density lipoprotein cholesterol
LDL-c	low-density lipoprotein cholesterol
SAP	Statistical analysis plan
CONSORT	Consolidated Standards of Reporting Trials

BMI	Body Mass Index
SD	Standard Deviation
MAR	Missing at Random
MNAR	Missing Not at Random
BOCF	Bast Observation Carried Forward
ID	Idenfitication number
CNPq	Brazilian National Council for Scientific and Technological Development
CAPES	Coordination for the Improvement of Higher Education Personnel
IRB	Institutional review board

20. References

1. Chan AW, Tetzlaff JM, Altman DG, Laupacis A, Gøtzsche PC, Krleža-Jerić K, et al. SPIRIT 2013 statement: Defining standard protocol items for clinical trials. Ann Intern Med. 2013 Feb 5;158(3):200–7.
2. Gonçalves DM, Stein AT, Kapczinski F. Avaliação de desempenho do Self-Reporting Questionnaire como instrumento de rastreamento psiquiátrico: um estudo comparativo com o Structured Clinical Interview for DSM-IV-TR. Cad Saude Publica. 2008 Feb;24(2):380–90.
3. Ciconelli RM, Ferraz MB, Ferraz MB, Santos W, Meinão I, Quaresma MR. Brazilian-Portuguese version of the SF-36. A reliable and valid quality of life outcome measure. Rev bras reumatol. 1999;39(3):143–50.
4. Teló GH, Iorra F de Q, Velho BS, Sparrenberger K, Schaan BD. Validation to Brazilian Portuguese of the Self-Care Inventory-revised for adults with type 2 diabetes. Arch Endocrinol Metab [Internet]. 2020 Mar 1 [cited 2024 Oct 2];64(2):190. Available from: /pmc/articles/PMC10118953/

5. Luft CDB, Sanches S de O, Mazo GZ, Andrade A. Versão brasileira da Escala de Estresse Percebido: tradução e validação para idosos. Rev Saude Publica. 2007 Aug;41(4):606–15.
6. BIOSTATVN. Statistics and Sample Size Pro software. 2023.
7. Fulmer R, Joerin A, Gentile B, Lakerink L, Rauws M. Using Psychological Artificial Intelligence (Tess) to Relieve Symptoms of Depression and Anxiety: Randomized Controlled Trial. JMIR Ment Health [Internet]. 2018 Oct 1 [cited 2023 Nov 13];5(4). Available from: /pmc/articles/PMC6315222/
8. Gamble C, Krishan A, Stocken D, Lewis S, Juszczak E, Doré C, et al. Guidelines for the Content of Statistical Analysis Plans in Clinical Trials. JAMA [Internet]. 2017 Dec 19 [cited 2023 Nov 13];318(23):2337–43. Available from: https://jamanetwork.com/journals/jama/fullarticle/2666509
9. Schulz KF, Altman DG, Moher D. CONSORT 2010 statement: Updated guidelines for reporting parallel group randomized trials. Ann Intern Med. 2010 Jun 1;152(11):726–32.
10. Committee for Medicinal Products for Human Use (CHMP) from the European Medicines Evaluation Agency. Guideline on Missing Data in Confirmatory Clinical Trials [Internet]. 2009. Available from: www.ema.europa.eu

Appendix A: SPIRIT Checklist

Section/item	Item No	Description	Location where item is reported
**Administrative information**			
Title	1	Descriptive title identifying the study design, population, interventions, and, if applicable, trial acronym	p. 03
Trial registration	2a	Trial identifier and registry name. If not yet registered, name of intended registry	p. 03
	2b	All items from the World Health Organization Trial Registration Data Set	p. 03
Protocol version	3	Date and version identifier	p. 03
Funding	4	Sources and types of financial, material, and other support	p. 20
Roles and responsibilities	5a	Names, affiliations, and roles of protocol contributors	p. 03
	5b	Name and contact information for the trial sponsor	p. 03
	5c	Role of study sponsor and funders, if any, in study design; collection, management, analysis, and interpretation of data; writing of the report; and the decision to submit the report for publication, including whether they will have ultimate authority over any of these activities	p. 21
	5d	Composition, roles, and responsibilities of the coordinating centre, steering committee, endpoint adjudication committee, data management team, and other individuals or groups overseeing the trial, if applicable (see Item 21a for data monitoring committee)	p. 21
**Introduction**			
Background and rationale	6a	Description of research question and justification for undertaking the trial, including summary of relevant studies (published and unpublished) examining benefits and harms for each intervention	p. 03
	6b	Explanation for choice of comparators	p. 03
Objectives	7	Specific objectives or hypotheses	p. 03
Trial design	8	Description of trial design including type of trial (eg, parallel group, crossover, factorial, single group), allocation ratio, and framework (eg, superiority, equivalence, noninferiority, exploratory)	p. 03

**Methods: Participants, interventions, and outcomes**			
Study setting	9	Description of study settings (eg, community clinic, academic hospital) and list of countries where data will be collected. Reference to where list of study sites can be obtained	p. 04
Eligibility criteria	10	Inclusion and exclusion criteria for participants. If applicable, eligibility criteria for study centres and individuals who will perform the interventions (eg, surgeons, psychotherapists)	p. 04
Interventions	11a	Interventions for each group with sufficient detail to allow replication, including how and when they will be administered	p. 06-11
	11b	Criteria for discontinuing or modifying allocated interventions for a given trial participant (eg, drug dose change in response to harms, participant request, or improving/worsening disease)	p. 06-11
	11c	Strategies to improve adherence to intervention protocols, and any procedures for monitoring adherence (eg, drug tablet return, laboratory tests)	p. 20
	11d	Relevant concomitant care and interventions that are permitted or prohibited during the trial	p. 06-11
Outcomes	12	Primary, secondary, and other outcomes, including the specific measurement variable (eg, systolic blood pressure), analysis metric (eg, change from baseline, final value, time to event), method of aggregation (eg, median, proportion), and time point for each outcome. Explanation of the clinical relevance of chosen efficacy and harm outcomes is strongly recommended	p. 12-13, p. 17
Participant timeline	13	Time schedule of enrolment, interventions (including any run-ins and washouts), assessments, and visits for participants. A schematic diagram is highly recommended (see Figure)	p. 13-15
Sample size	14	Estimated number of participants needed to achieve study objectives and how it was determined, including clinical and statistical assumptions supporting any sample size calculations	p. 16
Recruitment	15	Strategies for achieving adequate participant enrolment to reach target sample size	p. 04-05
**Methods: Assignment of interventions (for controlled trials)**			
Allocation:			
Sequence generation	16a	Method of generating the allocation sequence (eg, computer-generated random numbers), and list of any factors for stratification. To reduce predictability of a random sequence, details of any planned restriction (eg, blocking) should be	p. 06

		provided in a separate document that is unavailable to those who enrol participants or assign interventions	
Allocation concealment mechanism	16b	Mechanism of implementing the allocation sequence (eg, central telephone; sequentially numbered, opaque, sealed envelopes), describing any steps to conceal the sequence until interventions are assigned	p. 06
Implementation	16c	Who will generate the allocation sequence, who will enrol participants, and who will assign participants to interventions	p. 06
Blinding (masking)	17a	Who will be blinded after assignment to interventions (eg, trial participants, care providers, outcome assessors, data analysts), and how	p. 06
	17b	If blinded, circumstances under which unblinding is permissible, and procedure for revealing a participant's allocated intervention during the trial	p. 06
**Methods: Data collection, management, and analysis**			
Data collection methods	18a	Plans for assessment and collection of outcome, baseline, and other trial data, including any related processes to promote data quality (eg, duplicate measurements, training of assessors) and a description of study instruments (eg, questionnaires, laboratory tests) along with their reliability and validity, if known. Reference to where data collection forms can be found, if not in the protocol	p. 13, p. 15-16, p. 20
	18b	Plans to promote participant retention and complete follow-up, including list of any outcome data to be collected for participants who discontinue or deviate from intervention protocols	p. 16
Data management	19	Plans for data entry, coding, security, and storage, including any related processes to promote data quality (eg, double data entry; range checks for data values). Reference to where details of data management procedures can be found, if not in the protocol	p. 20
Statistical methods	20a	Statistical methods for analysing primary and secondary outcomes. Reference to where other details of the statistical analysis plan can be found, if not in the protocol	p. 17
	20b	Methods for any additional analyses (eg, subgroup and adjusted analyses)	p. 19
	20c	Definition of analysis population relating to protocol non-adherence (eg, as randomised analysis), and any statistical methods to handle missing data (eg, multiple imputation)	p. 17-18
**Methods: Monitoring**			
Data monitoring	21a	Composition of data monitoring committee (DMC); summary of its role and reporting structure; statement of whether it is independent from the sponsor and competing	p. 21

		interests; and reference to where further details about its charter can be found, if not in the protocol. Alternatively, an explanation of why a DMC is not needed	
	21b	Description of any interim analyses and stopping guidelines, including who will have access to these interim results and make the final decision to terminate the trial	p. 17
Harms	22	Plans for collecting, assessing, reporting, and managing solicited and spontaneously reported adverse events and other unintended effects of trial interventions or trial conduct	p. 20
Auditing	23	Frequency and procedures for auditing trial conduct, if any, and whether the process will be independent from investigators and the sponsor	p. 21
**Ethics and dissemination**			
Research ethics approval	24	Plans for seeking research ethics committee/institutional review board (REC/IRB) approval	p. 21
Protocol amendments	25	Plans for communicating important protocol modifications (eg, changes to eligibility criteria, outcomes, analyses) to relevant parties (eg, investigators, REC/IRBs, trial participants, trial registries, journals, regulators)	p. 21
Consent or assent	26a	Who will obtain informed consent or assent from potential trial participants or authorised surrogates, and how (see Item 32)	p. 05
	26b	Additional consent provisions for collection and use of participant data and biological specimens in ancillary studies, if applicable	N/A - No biological specimens were collected as part of the trial
Confidentiality	27	How personal information about potential and enrolled participants will be collected, shared, and maintained in order to protect confidentiality before, during, and after the trial	p. 20
Declaration of interests	28	Financial and other competing interests for principal investigators for the overall trial and each study site	p. 22
Access to data	29	Statement of who will have access to the final trial dataset, and disclosure of contractual agreements that limit such access for investigators	p. 21
Ancillary and post-trial care	30	Provisions, if any, for ancillary and post-trial care, and for compensation to those who suffer harm from trial participation	N/A – No provisions for post-trial care or compensation are anticipated
Dissemination policy	31a	Plans for investigators and sponsor to communicate trial results to participants, healthcare professionals, the public, and other relevant groups (eg, via publication,	p. 21

		reporting in results databases, or other data sharing arrangements), including any publication restrictions	
	31b	Authorship eligibility guidelines and any intended use of professional writers	p. 21
	31c	Plans, if any, for granting public access to the full protocol, participant-level dataset, and statistical code	p. 21
**Appendices**			
Informed consent materials	32	Model consent form and other related documentation given to participants and authorised surrogates	p. 21
Biological specimens	33	Plans for collection, laboratory evaluation, and storage of biological specimens for genetic or molecular analysis in the current trial and for future use in ancillary studies, if applicable	N/A - No biological specimens were collected as part of the trial

600

Appendix B: Description of the 28 daily messages repeated every 4 weeks in Brazilian Portuguese

No	Content
1	Separe um tempo do seu dia para você. Quem sabe vá ler um livro, revista ou jornal
2	Faça um ato de gentileza consigo mesmo hoje, vá e tome um banho relaxante ou tome uma xícara de chá quente agora
3	É importante ter momentos de relaxamento e atividades de lazer em sua rotina diária. Procure encontrar um tempo no seu dia para fazer algo que você gosta
4	É importante manter contato com amigos e familiares, inclusive por meio de chamadas telefônicas regulares. O que acha de ligar para alguém querido agora?
5	Que tal estimular a sua mente resolvendo problemas de memória? Eu posso lhe ajudar com isso com um jogo de perguntas. Caso tenha interesse basta dizer: “Alexa abrir Meu Quiz”
6	Aproveite o sol da manhã cedo ou no final da tarde para obter um pouco de vitamina D
7	Que tal fazer uma pausa agora e rever fotos antigas de momentos especiais? Reviver memórias positivas é importante e pode fazer bem
8	Que tal assistir algum filme ou novela que lhe traga alegria ou que você goste?
9	Que tal experimentar uma receita de culinária saudável hoje?
10	Ao se alimentar, opte por alimentos integrais, como pães e cereais, ao invés de alimentos com carboidratos refinados

11	Que tal se desafiar e tentar aumentar o consumo de vegetais, como alface, couve e espinafre essa semana? São alimentos ricos em fibras e nutrientes que podem ajudar com o controle da diabetes
12	É importante limitar o consumo de alimentos processados como salgadinhos e alimentos congelados
13	Você pode reduzir o consumo de sal utilizado nos alimentos substituindo-o por outros temperos naturais como ervas, especiarias e pimenta do reino. Que tal tentar fazer um prato com menos sal hoje?
14	Você pode substituir sobremesas açucaradas por opções mais saudáveis como frutas frescas ou iogurte natural
15	Você pode substituir sobremesas açucaradas por opções mais saudáveis como frutas frescas ou iogurte natural
16	Faça refeições equilibradas, incluindo uma fonte de proteína ou carne, vegetais, poucos carboidratos e poucas gorduras
17	Leia os rótulos dos alimentos e evite aqueles que contêm muito açúcares e ingredientes não saudáveis
18	Faça refeições caseiras sempre que possível para ter controle dos ingredientes e do preparo
19	Sempre que verificar o nível de glicose no sangue ou tiver um episódio de hipoglicemia, anote em um caderninho com a data e o valor
20	Caso tenha um episódio de hipoglicemia lembre-se que basta uma colher de sopa de açúcar para reverter. Cuidado pois o excesso de açúcar também pode ser um problema
21	Tenha um plano de emergência em caso de hipoglicemia, lembre-se de levar algo para comer quando sair de casa

22	Mantenha um estoque adequado de medicamentos e suprimentos relacionados ao diabetes, como fitas de HGT, medicações e agulhas de insulina para evitar interrupções no tratamento
23	Que tal aprender técnicas de relaxamento, como respiração profunda e meditação, para lidar com o estresse? Posso te ajudar com isso, basta solicitar: Alexa, abra a skill de meditação
24	Às vezes é normal sentir que ter diabetes é um peso e essas emoções fazem parte do processo de aprender a lidar com a doença. Dê atenção à sua saúde mental e emocional
25	O diabetes é uma condição controlável; com o tratamento adequado, você pode manter os níveis de glicose no sangue sob controle e levar uma vida boa e normal
26	Busque apoio de familiares, amigos e grupos de apoio para compartilhar experiências, dicas e desafios relacionados com o tratamento de diabetes. Ter pessoas que entendam e apoiem você pode fazer toda a diferença
27	O objetivo do tratamento do diabetes é melhorar sua qualidade de vida e bem-estar geral. Concentre-se em cuidar de si mesmo e fazer escolhas saudáveis que tragam benefícios a longo prazo para sua vida
28	Mesmo com o diagnóstico de diabetes você não precisa abrir mão de todas as coisas que ama e de comer as coisas que gosta

6.8 Appendix C: Description of the 7 sets of 14 messages repeated every week translated in Brazilian Portuguese

No	Content
1	Se possível, realize uma sessão de alongamento
	Praticar atividades físicas de intensidade moderada por 30 minutos por dia é o suficiente para deixar de ser sedentário. Esses 30 minutos podem ser divididos em períodos de 10 minutos. Procure sempre se manter ativo.
2	Os sintomas mais comuns de hipoglicemia são: tremores, tonturas, suor intenso, desmaio. Lembre-se de sempre cuidar a presença desses sintomas.
	Procure utilizar as doses corretas de insulina e sempre aplicar no horário orientado pela sua equipe médica
3	Lembre-se de tomar cuidado com seus pés, cuide para não se machucar e sempre verifique a presença de lesões ou feridas. Cuide seus calçados e sempre confira se estão adequados para uso.
	Mantenha acompanhamento médico regular, faça seu HGT conforme orientação da sua equipe médica e procure usar corretamente as medicações prescritas
4	Evite beber café e chimarrão após as 17 horas; essas bebidas possuem cafeína e podem atrapalhar o seu sono
	Além disso, diminuir o uso de dispositivos eletrônicos com telas algumas horas antes de dormir é importante. Todas essas atitudes irão ajudar você a dormir melhor
5	Lembre-se de beber água regularmente
	Evitar cochilos após as 17 horas pode fazer com que seja mais fácil dormir à noite
6	Está se sentindo sozinho? Que tal mandar uma mensagem ou telefonar para algum amigo ou familiar agora?
	Lembre-se de criar metas diárias alcançáveis e de comemorar pequenas conquistas
7	Vamos meditar agora? Caso tenha interesse, basta pedir pela skill de meditação

	Escutar uma música que você goste pode estimular o ânimo e o bem-estar. Posso tocar qualquer música para você, basta pedir: “Alexa, toque música"

609
