# Supplementary material for: Interactive Virtual Assistant for Health Promotion Among Older Adults With Type 2 Diabetes: The IVAM-ED Randomized Clinical Trial
Source: JAMA Netw Open. 2026 Jan 23;9(1):e2553508. doi: 10.1001/jamanetworkopen.2025.53508 (PMC12831153; doi:10.1001/jamanetworkopen.2025.53508)
Supplement: Supplement 2. — eAppendix 1. Eligibility Criteria eAppendix 2. Study Outcomes eTable 1. Standardized Differences Between Study Groups for Demographic and Clinical Characteristics at Baseline eTable 2. Additional Demographic and Clinical Characteristics of the Enrolled Participants eTable 3. Baseline Demographic and Clinical Characteristics of Patients With and Without Missing Outcome Data at 12-Week Follow-Up eTable 4. Additional Secondary Outcomes at 12-Week Follow-Up eTable 5. Sensitivity Analysis of Primary and Secondary Outcomes at 12-Week Follow-Up Excluding Participants With Missing Outcome Data eTable 6. Sensitivity Analysis of Primary and Secondary Outcomes at 12-Week Follow-Up Using BOCF for Missing Outcome Data eTable 7. Reported Deaths During the Study Period [file jamanetwopen-e2553508-s002.pdf]

Supplemental Online Content

Matzenbacher LS, da Costa FL, de Barros LGB, et al. Interactive virtual assistant for health promotion among older adults with type 2 diabetes: the IVAM-ED randomized clinical trial. *JAMA Netw Open*. 2026;9(1):e2553508.
doi:10.1001/jamanetworkopen.2025.53508

eAppendix 1. Eligibility Criteria
eAppendix 2. Study Outcomes
eTable 1. Standardized Differences Between Study Groups for Demographic and Clinical Characteristics at Baseline
eTable 2. Additional Demographic and Clinical Characteristics of the Enrolled Participants
eTable 3. Baseline Demographic and Clinical Characteristics of Patients With and Without Missing Outcome Data at 12-Week Follow-Up
eTable 4. Additional Secondary Outcomes at 12-Week Follow-Up
eTable 5. Sensitivity Analysis of Primary and Secondary Outcomes at 12-Week Follow-Up Excluding Participants With Missing Outcome Data
eTable 6. Sensitivity Analysis of Primary and Secondary Outcomes at 12-Week Follow-Up Using BOCF for Missing Outcome Data
eTable 7. Reported Deaths During the Study Period

This supplemental material has been provided by the authors to give readers additional information about their work.

© 2026 Matzenbacher LS et al. *JAMA Network Open*.

eAppendix 1. Eligibility Criteria

Inclusion criteria

Participants were eligible for inclusion if all of the following criteria were met:

1. Being 65 years of age or older at the time of enrollment;
2. Having type 2 diabetes;
3. Residing in the city of Porto Alegre or metropolitan region (Southern Brazil);
4. Having availability of Wi-Fi connection at their place of residence;
5. Availability to participate in two face-to-face evaluations at the Clinical Research Center of Hospital São Lucas as part of the trial;
6. Acceptance of one home visit by a research team member for device installation if randomized to the intervention group.

Exclusion criteria

Participants were not eligible for inclusion if any of the following criteria apply:

1. Already having an interactive virtual assistance device at the time of enrollment;
2. Presence of significant cognitive impairment, defined as a cognitive impairment severe enough to prevent adequate interaction with outcome assessors;
3. Presence of significant hearing impairment, defined as severe enough to prevent adequate interaction with outcome assessors;
4. Residing in regions of difficult access, defined as regions where a home visit (necessary for device installation for those in the intervention group) could place a research team member at risk.

© 2026 Matzenbacher LS et al. *JAMA Network Open*.

eAppendix 2. Study Outcomes

**Primary outcome**	
Mental distress	Mental distress was assessed using the Brazilian version of the Self-Reporting Questionnaire 20 (SRQ-20). The SRQ-20 is a 20-item survey measuring symptoms of common mental health disorders experienced in the past month. Scores range from 0 to 20, with higher scores indicating greater levels of mental distress
**Secondary outcomes**	
Quality of life	Quality of life was assessed using the Brazilian version of the 36-Item Short Form Health Survey (SF-36), which measures general quality of life across eight domains. Quality of life is reported as an average score across these domains, with a total score ranging from 0 to 100, where higher scores indicate better quality of life.
Perceived stress	Perceived stress was assessed using the Brazilian version of the Perceived Stress Scale (PSS). The PSS is a 14-item questionnaire measuring participants' perceptions of stress over the past month. Scores range from 0 to 56, with higher scores indicating greater levels of perceived stress.
Adherence to diabetes self-care behaviors	Adherence to diabetes self-care behaviors was assessed using the Brazilian version of the Self-Care Inventory – Revised (SCI-R) for patients with type 2 diabetes. The SCI-R is an 11-item survey that measures adherence to diabetes-related self-care behaviors over the past one to two months. Scores range from 11 to 55, with higher scores indicating greater adherence to diabetes self-care behaviors.
Glycemic control	Glycemic control was evaluated by measuring glycated hemoglobin (HbA1c), which reflects average blood glucose levels over the past three months. All measurements were conducted in the central laboratory of the clinical research center at Hospital São Lucas. Results are reported as percentages, representing the HbA1c fraction, or as mmol/mol.
**Additional secondary outcomes**	
Systolic and diastolic blood pressure	Systolic and diastolic blood pressure were reported as the mean of three repeated measurements taken using an appropriately sized cuff based on arm circumference.
Lipid profile	Lipid profile was evaluated by measuring total cholesterol, LDL cholesterol, HDL cholesterol, and triglycerides after eight hours of fasting. All measurements were conducted in the central laboratory of the clinical research center at Hospital São Lucas.

© 2026 Matzenbacher LS et al. *JAMA Network Open*.

eTable 1. Standardized Differences Between Study Groups for Demographic and Clinical Characteristics at Baseline

Characteristic	Standardized differences between study groups
**Age, y**	-0.22
**Weight, kg**	-0.07
**BMI, kg/m^2**	-0.20
**Sex**	
Female	-0.11
Male	0.11
**Educational level**	
No qualification	0.25
Primary education	0.15
Secondary education	-0.41
University degree or higher	0.00
**Educational level[a], y**	0.27
**Monthly income[b], No. of Brazilian minimum wages**	0.16
**Diabetes duration, y**	-0.07
**Diabetes complications**	
Macrovascular	-0.04
Microvascular	0.25
**Diabetes medications**	
Oral hypoglycemic agents	
Metformin	-0.32
Sulfonylureas	-0.08
SGLT-2 inhibitors	0.25
DPP-4 inhibitors	-0.23
Glitazones	-0.38
GLP-1 receptor agonists	-0.27
Insulin	0.14
**HbA_{1c} level, %**	-0.10
**HbA_{1c} level, mmol/mol**	-0.10

Continued on next page

eTable 1. Standardized Differences Between Study Groups for Demographic and Clinical Characteristics at Baseline *(continued)*

**Characteristic**	**Standardized differences between study groups**
**Mental Health**	
Suicidal thoughts	-0.12
Depression	-0.07
Anxiety	-0.30
Bipolar disorder	-0.11
**Blood pressure[c], mmHg**	
Systolic	0.03
Diastolic	-0.16
**Plasma lipid level, mg/dL**	
Total cholesterol	-0.09
LDL cholesterol	-0.06
HDL cholesterol	0.11
Triglycerides	-0.25
**MMSE score**	0.30
**SRQ-20 score**	-0.17
**SCI-R score**	0.21
**PSS score**	0.29
**SF-36 score**	-0.03

Standardized difference corresponds to Cohen's *d* for continuous variables and Cohen's *h* for categorical variables. Abbreviation: BMI, body mass index (calculated as weight in kilograms divided by height in meters squared); SGLT2, sodium-glucose transport protein 2; DPP-4, dipeptidyl peptidase-4; HbA_{1c}, glycated hemoglobin; LDL, low-density lipoprotein; HDL, high-density lipoprotein; MMSE, mini-mental state examination; SRQ, self-report questionnaire; SCI-R, self-care inventory-revised (version for type 2 diabetes); PSS, perceived stress scale; SF-36, short form 36. [a]Number of complete years of study. [b]Monthly income expressed as the number of Brazilian minimum wages. Each minimum wage corresponds to a monthly income of R$1320.00 (equivalent to USD 257.35 or EUR 237.86, based on the exchange rate at the time of data collection). For reference, a mean income of 2.5 minimum wages corresponds to R$3300.00 (USD 643 or EUR 595) per month, and 2.1 minimum wages corresponds to R$2770.00 (USD 540 or EUR 500) per month. [c]Mean of three repeated measures of blood pressure with an appropriate cuff considering the arm circumference.

© 2026 Matzenbacher LS et al. *JAMA Network Open*.

eTable 2. Additional Demographic and Clinical Characteristics of the Enrolled Participants

Characteristic	Usual care (n = 56)	Smart speaker (n = 56)
**Age**		
Median (IQR), y	72.5 (67.3 – 76.0)	71.5 (68.0 – 77.8)
65 to 70 years, No (%)	24 (42.9)	20 (35.7)
70 to 75 years, No (%)	12 (21.4)	18 (32.1)
75 to 80 years, No (%)	13 (23.2)	12 (21.4)
≥ 80 years, No (%)	7 (12.5)	6 (10.7)
**Diabetes duration**		
Median (IQR), y	15.0 (6.3 – 24.8)	15.5 (7.0 – 25.0)
Duration ≥ 10 years, No (%)	38 (67.9)	39 (69.6)
**Insulin regimen, No (%)**		
Basal	16 (28.6)	15 (26.8)
Basal-bolus	12 (21.4)	17 (30.4)
**HbA_{1c} level**		
Median (IQR), %	7.8 (6.6 – 7.8)	7.7 (6.9 – 8.6)
Median (IQR), mmol/mol	61.8 (48.9 – 72.4)	60.7 (52.2 – 70.5)
**FPG, mean (SD), mg/dL**	119.9 (40.1)	149.8 (57.2)
**History of smoking**	31 (55.3)	29 (51.8)
**History of alcoholism**	6 (10.7)	13 (23.2)
**Medications in use**		
Number of medications, mean (SD)	8.8 (2.6)	9.3 (2.7)
Number of antidiabetic medications, mean (SD)	2.2 (1.1)	2.2 (1.1)
Benzodiazepines, No (%)	1 (1.8)	1 (1.8)
SSRIs, No (%)	10 (17.9)	11 (19.6)
SNRIs, No (%)	4 (7.1)	7 (12.5)
Tricyclic antidepressants, No (%)	4 (7.1)	3 (5.4)
Atypical antidepressants, No (%)	2 (3.6)	3 (5.4)
Mood stabilizer, No (%)	6 (10.7)	2 (3.6)
Statin, No (%)	47 (83.9)	49 (87.5)
Antihypertensive drugs, No (%)	50 (89.3)	55 (98.2)
Platelet inhibitor agents, No (%)	21 (37.5)	21 (37.5)

Continued on next page

© 2026 Matzenbacher LS et al. *JAMA Network Open*.

eTable 2. Additional Demographic and Clinical Characteristics of the Enrolled Participants *(continued)*

**Characteristic**	**Usual care (n = 56)**	**Smart speaker (n = 56)**
**Comorbidities**		
CKD, No (%)	7 (12.5)	13 (23.2)
Cancer, No (%)	8 (14.3)	14 (25.0)
Stroke, No (%)	17 (30.4)	14 (25.0)
Coronary artery disease, No (%)	21 (37.5)	22 (39.3)
Myocardial infarction, No (%)	11 (19.6)	6 (10.7)
Heart failure, No (%)	7 (12.5)	8 (14.3)
Hypertension, No (%)	50 (89.3)	54 (96.4)
Dyslipidemia, No (%)	50 (89.3)	51 (91.1)
Hypothyroidism, No (%)	9 (16.1)	13 (23.2)
Osteoporosis, No (%)	5 (8.9)	5 (8.9)
**Gait speed, mean (SD), s**	5.42 (2.3)	5.51 (1.6)
**Sit-to-stand[a], mean (SD)**	6.2 (2.8)	5.5 (2.7)
**Up and go, mean (SD), s**	17.44 (8.0)	17.73 (6.8)
**Handgrip strength[b], mean (SD), kgf**	18.63 (7.4)	17.77 (8.2)
**Katz ADL score, mean (SD)**	0.6 (1.0)	0.7 (0.9)
**Lawton IADL score, mean (SD)**	23.1 (4.1)	21.9 (4.8)

Abbreviation: IQR, interquartile range (quartile one to quartile three); HbA_{1c}, glycated hemoglobin; FPG, fasting plasma glucose; SD, standard deviation; SSRIs, selective serotonin reuptake inhibitors; SNRIs, serotonin and norepinephrine reuptake inhibitors; CKD, chronic kidney disease; ADL, activity of daily living; IADL, instrumental activity of daily living. [a]Number of completed cycles of sit-to-stand in 30 seconds. [b]Mean of three repeated measures in the non-dominant arm.

© 2026 Matzenbacher LS et al. *JAMA Network Open*.

eTable 3. Baseline Demographic and Clinical Characteristics of Patients With and Without Missing Outcome Data at 12-Week Follow-Up

Characteristic	Patients without missing outcome data (n = 103)	Patients with missing outcome data (n = 9)
**Age, mean (SD), y**	72.6 (5.8)	72.0 (5.4)
**BMI, mean (SD), kg/m^2**	30.6 (6.2)	30.8 (4.5)
**Sex**		
Female	63 (61.2)	8 (88.9)
Male	40 (38.8)	1 (11.1)
**Educational level[a], mean (SD), y**	6.9 (4.1)	7.0 (4.1)
**Monthly income[b], mean (SD), No. of Brazilian minimum wages**	2.3 (2.5)	2.2 (1.6)
**Diabetes duration, mean (SD), y**	17.3 (11.6)	11.3 (10.7)
**HbA_{1c} level, mean (SD), %**	8.0 (1.5)	6.9 (1.0)
**Mental Health**		
Depression	33 (32.0)	7 (77.8)
Anxiety	32 (31.1)	6 (66.7)
**MMSE score, mean (SD)**	24.3 (4.3)	22.7 (4.2)
**SRQ-20 score, mean (SD)**	7.4 (4.9)	8.8 (5.5)
**SCI-R score, mean (SD)**	36.0 (6.3)	34.0 (8.2)
**PSS score, mean (SD)**	21.8 (10.2)	26.7 (26.0)
**SF-36 score, mean (SD)**	50.8 (21.4)	47.1 (26.0)

Abbreviation: SD, standard deviation; BMI, body mass index (calculated as weight in kilograms divided by height in meters squared); MMSE, mini-mental state examination; SRQ, self-report questionnaire; SCI-R, self-care inventory-revised (version for type 2 diabetes); PSS, perceived stress scale; SF-36, short form 36. [a]Number of complete years of study. [b]Monthly income expressed as the number of Brazilian minimum wages. Each minimum wage corresponds to a monthly income of R$1320.00 (equivalent to USD 257.35 or EUR 237.86, based on the exchange rate at the time of data collection). For reference, a mean income of 2.5 minimum wages corresponds to R$3300.00 (USD 643 or EUR 595) per month, and 2.1 minimum wages corresponds to R$2770.00 (USD 540 or EUR 500) per month.

© 2026 Matzenbacher LS et al. *JAMA Network Open*.

eTable 4. Additional Secondary Outcomes at 12-Week Follow-Up

Outcome	Usual care (n = 56)	Smart speaker (n = 56)	Mean difference (95% CI)	Effect size[c]	P value[d]
**SBP**					
At 12 w, baseline-adjusted mean (SE)[a], mmHg	133.32 (2.02)	135.88 (2.06)	2.55 (-8.09 to 2.98)	0.18	0.365
At 12 w, fully adjusted mean (SE)[b], mmHg	133.50 (2.01)	135.70 (2.05)	2.21 (-3.37 to 7.78)	0.16	0.437
**DBP**					
At 12 w, baseline-adjusted mean (SE)[a], mmHg	76.81 (1.13)	76.17 (1.08)	-0.64 (-3.82 to 2.53)	0.08	0.689
At 12 w, fully adjusted mean (SE)[b], mmHg	77.03 (1.15)	75.95 (1.10)	-1.08 (-4.34 to 2.19)	0.14	0.515
**Total cholesterol**					
At 12 w, baseline-adjusted mean (SE)[a], mg/dL	169.26 (4.54)	175.60 (4.61)	6.34 (-6.47 to 19.15)	0.20	0.331
At 12 w, fully adjusted mean (SE)[b], mg/dL	169.99 (4.58)	174.87 (4.68)	4.88 (-8.46 to 18.21)	0.15	0.473
**LDL cholesterol**					
At 12 w, baseline-adjusted mean (SE)[a], mg/dL	89.69 (3.62)	93.93 (3.67)	4.24 (-6.06 to 14.54)	0.16	0.419
At 12 w, fully adjusted mean (SE)[b], mg/dL	89.66 (3.67)	93.66 (3.74)	3.70 (-7.07 to 14.47)	0.14	0.500
**HDL cholesterol**					
At 12 w, baseline-adjusted mean (SE)[a], mg/dL	49.73 (1.19)	53.12 (1.30)	3.38 (0.13 to 6.64)	0.41	0.041
At 12 w, fully adjusted mean (SE)[b], mg/dL	49.77 (1.20)	53.07 (1.28)	3.30 (-0.42 to 7.02)	0.41	0.082
**Triglycerides**					
At 12 w, baseline-adjusted mean (SE)[a], mg/dL	193.25 (14.01)	186.35 (15.24)	-6.90 (-43.55 to 29.76)	0.07	0.711
At 12 w, fully adjusted mean (SE)[b], mg/dL	195.54 (13.91)	184.05 (14.76)	-11.49 (-46.67 to 23.69)	0.12	0.521

Abbreviation: SBP, systolic blood pressure; SD, standard deviation; SE, standard error; DBP, diastolic blood pressure; LDL, low density lipoprotein; HDL, high density lipoprotein. [a]Baseline data for the outcome were included as covariate in the baseline-adjusted model. [b]Baseline data for the outcome variable, age, sex, education, income, and mini-mental state examination score were included as covariates in the fully adjusted model. [c]Standardized mean difference (SMD). [d]Between-group pairwise comparisons using analysis of covariance. The model included all enrolled participants, with missing data replaced using multiple imputation. Statistical significance was considered when $p < 0.05$.

© 2026 Matzenbacher LS et al. *JAMA Network Open*.

eTable 5. Sensitivity Analysis of Primary and Secondary Outcomes at 12-Week Follow-Up Excluding Participants With Missing Outcome Data

	**Usual care (n = 51)**	**Smart speaker (n = 52)**	**Mean difference (95% CI)**	**Effect size[f]**	**P value[g]**
**SRQ-20 score at 12 w, fully adjusted mean (SE)[a,b]**	7.58 (0.43)	6.09 (0.43)	-1.50 (-2.77 to -0.22)	0.49	0.022
**Secondary outcomes at 12 w, fully adjusted mean (SE)[b]**					
HbA_{1c} level, %	8.08 (0.13)	7.61 (0.13)	-0.47 (-0.84 to -0.10)	0.51	0.012
HbA_{1c} level, mmol/mol	64.85 (1.45)	59.71 (1.44)	-5.14 (-9.14 to -1.14)	0.51	0.012
SCI-R score[c]	36.44 (0.64)	39.82 (0.63)	3.38 (1.60 to 5.16)	0.75	<0.001
PSS score[d]	22.06 (1.18)	18.62 (1.17)	-3.44 (0.11 to 6.77)	0.41	0.043
SF-36 score[e]	48.74 (1.97)	58.60 (1.95)	9.86 (3.92 to 15.80)	0.71	0.001
SBP, mmHg	133.68 (2.02)	136.59 (1.98)	2.90 (-2.86 to 8.66)	0.21	0.320
DBP, mmHg	77.34 (1.12)	76.18 (1.09)	-1.17 (-4.34 to 2.01)	0.15	0.467
Total cholesterol, mg/dL	170.49 (4.63)	174.34 (4.59)	3.85 (-9.60 to 17.31)	0.12	0.571
LDL cholesterol, mg/dL	90.40 (3.70)	93.29 (3.67)	2.89 (-7.94 to 13.72)	0.11	0.598
HDL cholesterol, mg/dL	49.84 (1.16)	52.94 (1.15)	3.11 (-0.86 to 7.07)	0.39	0.123
Triglycerides, mg/dL	194.27 (13.28)	181.82 (13.15)	-12.45 (-47.22 to 22.33)	0.14	0.479

Abbreviation: SRQ, self-report questionnaire; SE, standard error; HbA_{1c}, glycated hemoglobin; SCI-R, self-care inventory-revised (version for type 2 diabetes); PSS, perceived stress scale; SF-36, short form 36; SBP, systolic blood pressure; DBP, diastolic blood pressure; LDL, low density lipoprotein; HDL, high density lipoprotein; CI, confidence interval. [a]Total score ranging from 0 to 20 points, with lower scores indicating less mental distress. [b]Baseline data for the outcome variable, age, sex, education, income, and mini-mental state examination score were included as covariates in the fully adjusted model. [c]Total score ranging from 11 to 55 points, with higher scores indicating a higher level of care related to diabetes. [d]Total score ranging from 0 to 56 points, with lower scores indicating a reduced level of stress. [e]Average of the total score on the eight domains of the SF-36 questionnaire, ranging from 0 to 100 and with higher scores indicating greater quality of life. [f]Standardized mean difference (SMD). [g]Between-group pairwise comparisons using analysis of covariance. The model included only those subjects who attended final assessment at 12 weeks (trial completers). Statistical significance was considered when $p < 0.05$.

© 2026 Matzenbacher LS et al. *JAMA Network Open*.

eTable 6. Sensitivity Analysis of Primary and Secondary Outcomes at 12-Week Follow-Up Using BOCF for Missing Outcome Data

	**Usual care (n = 56)**	**Smart speaker (n = 56)**	**Mean difference (95% CI)**	**Effect size[f]**	**P value[g]**
**SRQ-20 score at 12 w, fully adjusted mean (SE)[a,b]**	7.64 (0.40)	6.33 (0.40)	-1.31 (-2.48 to -0.14)	0.44	0.028
**Secondary outcomes at 12 w, fully adjusted mean (SE)[b]**					
HbA_{1c} level, %	7.98 (0.12)	7.56 (0.12)	-0.42 (-0.74 to -0.10)	0.48	0.011
HbA_{1c} level, mmol/mol	63.72 (1.29)	59.14 (1.29)	-4.58 (-8.10 to -1.06)	0.48	0.011
SCI-R score[c]	36.14 (0.61)	39.49 (0.61)	3.35 (1.62 to 5.07)	0.74	<0.001
PSS score[d]	22.42 (1.10)	19.24 (1.10)	-3.19 (-6.32 to -0.05)	0.39	0.046
SF-36 score[e]	48.73 (1.82)	57.64 (1.82)	8.91 (3.44 to 14.38)	0.66	0.002
SBP, mmHg	133.19 (1.78)	136.19 (1.77)	3.00 (-2.09 to 8.09)	0.23	0.245
DBP, mmHg	77.04 (0.99)	76.32 (0.98)	-0.72 (-3.53 to 2.10)	0.10	0.616
Total cholesterol, mg/dL	171.45 (4.10)	174.26 (4.10)	2.81 (-8.99 to 14.60)	0.09	0.638
LDL cholesterol, mg/dL	91.62 (3.32)	93.79 (3.32)	2.18 (-7.36 to 11.72)	0.09	0.652
HDL cholesterol, mg/dL	49.79 (1.03)	52.48 (1.03)	2.70 (-0.77 to 6.16)	0.36	0.126
Triglycerides, mg/dL	191.65 (11.74)	182.01 (11.74)	-9.64 (-43.10 to 20.83)	0.11	0.532

Abbreviation: BOCF, baseline observation carried forward; SRQ, self-report questionnaire; SE, standard error; HbA_{1c}, glycated hemoglobin; SCI-R, self-care inventory-revised (version for type 2 diabetes); PSS, perceived stress scale; SF-36, short form 36; SBP, systolic blood pressure; DBP, diastolic blood pressure; LDL, low density lipoprotein; HDL, high density lipoprotein; CI, confidence interval. [a]Total score ranging from 0 to 20 points, with lower scores indicating less mental distress. [b]Baseline data for the outcome variable, age, sex, education, income, and mini-mental state examination score were included as covariates in the fully adjusted model. [c]Total score ranging from 11 to 55 points, with higher scores indicating a higher level of care related to diabetes. [d]Total score ranging from 0 to 56 points, with lower scores indicating a reduced level of stress. [e]Average of the total score on the eight domains of the SF-36 questionnaire, ranging from 0 to 100 and with higher scores indicating greater quality of life. [f]Standardized mean difference (SMD). [g]Between-group pairwise comparisons using analysis of covariance. The model included all enrolled participants, with missing data repl aced using BOCF. Statistical significance was considered when $p < 0.05$.

© 2026 Matzenbacher LS et al. *JAMA Network Open*.

eTable 7. Reported Deaths During the Study Period

**Patient (age, sex)**	**Study group**	**Description / Cause of death**
65 years, female	Usual care	Urinary sepsis
68 years female	Smart speaker	Hemorrhagic stroke
73 years, female	Smart speaker	Heart failure decompensation

© 2026 Matzenbacher LS et al. *JAMA Network Open*.
