# Supplementary material for: Interactive Virtual Assistant for Health Promotion Among Older Adults With Type 2 Diabetes: The IVAM-ED Randomized Clinical Trial
Source: JAMA Netw Open. 2026 Jan 23;9(1):e2553508. doi: 10.1001/jamanetworkopen.2025.53508 (PMC12831153; doi:10.1001/jamanetworkopen.2025.53508)
Supplement: Supplement 3. — Data Sharing Statement [file jamanetwopen-e2553508-s003.pdf]

Data Sharing Statement

Matzenbacher. Interactive Virtual Assistant for Health Promotion Among Older Adults With Type 2 Diabetes. *JAMA Netw Open*. Published January 22, 2026.
doi:10.1001/jamanetworkopen.2025.53508

Data
Additional Information: ClinicalTrials.gov Identifier: NCT05329376.
Data available: Yes
Data types: Deidentified participant data
How to access data: Data can be made available upon reasonable request, with a justification and a data use agreement, by contacting the corresponding author (gabriela.telo@pucrs.br).
When available: beginning date: 12-31-2025, end date: 12-31-2027

Supporting Documents
Document types: Informed consent form
How to access documents: Available upon request to the corresponding author (gabriela.telo@pucrs.br)
When available: With publication

Additional Information
Who can access the data: Researchers with a manuscript proposal and justification
Types of analyses: Systematic reviews and meta-analyses
Mechanisms of data availability: Available upon request to the corresponding author (gabriela.telo@pucrs.br) after proposal approval and a signed data use agreement.
